# Glycyl-l-histidyl-l-lysine prevents copper- and zinc-induced protein aggregation and central nervous system cell death *in vitro*

**DOI:** 10.1093/mtomcs/mfae019

**Published:** 2024-04-10

**Authors:** Jin-Hong Min, Heela Sarlus, Robert A Harris

**Affiliations:** Department of Clinical Neuroscience, Karolinska Institutet, Center for Molecular Medicine, Karolinska University Hospital, S-171 76 Stockholm, Sweden; Department of Clinical Neuroscience, Karolinska Institutet, Center for Molecular Medicine, Karolinska University Hospital, S-171 76 Stockholm, Sweden; Department of Clinical Neuroscience, Karolinska Institutet, Center for Molecular Medicine, Karolinska University Hospital, S-171 76 Stockholm, Sweden

**Keywords:** GHK, cuproptosis, copper, zinc, aggregate, inflammation

## Abstract

Common features of neurodegenerative diseases are oxidative and inflammatory imbalances as well as the misfolding of proteins. An excess of free metal ions can be pathological and contribute to cell death, but only copper and zinc strongly promote protein aggregation. Herein we demonstrate that the endogenous copper-binding tripeptide glycyl-l-histidyl-l-lysine (GHK) has the ability to bind to and reduce copper redox activity and to prevent copper- and zinc-induced cell death *in vitro*. In addition, GHK prevents copper- and zinc-induced bovine serum albumin aggregation and reverses aggregation through resolubilizing the protein. We further demonstrate the enhanced toxicity of copper during inflammation and the ability of GHK to attenuate this toxicity. Finally, we investigated the effects of copper on enhancing paraquat toxicity and report a protective effect of GHK. We therefore conclude that GHK has potential as a cytoprotective compound with regard to copper and zinc toxicity, with positive effects on protein solubility and aggregation that warrant further investigation in the treatment of neurodegenerative diseases.

## Graphical Abstract

**Graphical Abstract:**
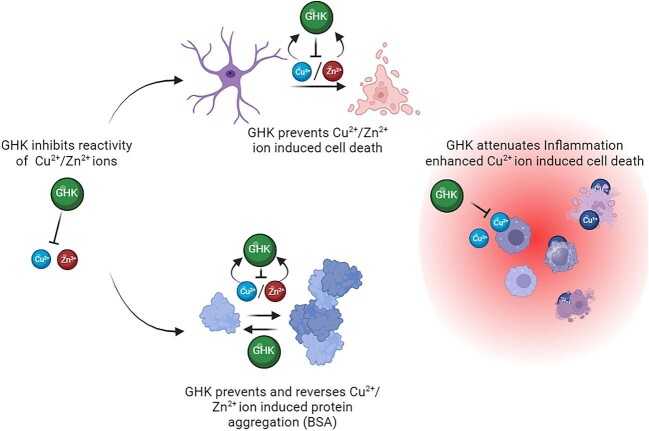
The protective effects of GHK on copper and zinc induced cell death, BSA aggregation and inflammation enhanced copper toxicity.

## Introduction

The brain is a highly metabolic organ that is significantly dependent on transition metals such as iron, zinc, and copper for homeostatic biological functions such as myelination, oxidative respiration, neurotransmitter synthesis, neurogenesis, and immune regulation.^1–4^ However, transition metal imbalance is a central feature of neurodegenerative diseases such as Alzheimer's disease (AD), Parkinson's disease (PD), Prion disease (PrD), amyotrophic lateral sclerosis (ALS), and neurological Wilson's disease.^5–10^ Interestingly, the involvement of copper and, to a lesser extent, zinc in protein aggregation and cytotoxicity exceeds that of iron or other metal ions.^11,12^

To understand this toxicity, we need to first understand the underlying mechanism of action. For instance, one facet of cytotoxicity of copper is directly linked to its physiological role as a cofactor in many enzymes, including cytochrome c oxidase complex 4 (COX4) for aerobic respiration, copper/zinc superoxide dismutase 1 (Cu/ZnSOD) for superoxide anion dismutation into peroxides, and ceruloplasmin as a ferroxidase for extracellular export of iron.^13^ This is due to the strong redox cycling ability of copper, and as a result of this, copper (and iron) is able to participate in reactive oxygen species (ROS) generation via Fenton chemistry, either when free or as sterically exposed ions on proteins. This can result in unregulated ROS production and tissue damage.^14,15^

Aside from ROS generation, another role of copper is in protein structural stability. One example includes SOD1, in which loss of copper and zinc decreases protein stability, with a 60-fold greater unfolding rate than the metalated enzyme, and also the copper chaperone ATOX1.^16,17^ This stability arises from the ability of copper to bind at mainly cysteine and histidine residues that can bind and stabilize proteins during and after polypeptide folding.^17^ However, in cases of excess copper exposure, pathological binding can occur, leading to the formation of aggregates that primarily consist of proteins enriched in either cysteine or histidine.^18,19^ In the case of cysteine-rich proteins, oxidative linking of cysteine-rich thiol groups together with intermolecular linkages by inner sphere electron transfer can lead to disulfide bridge formation between two proteins that persists even when copper is liberated.^20^ In the case of amyloid beta (Aβ) (an example of a protein containing multiple histidine-binding sites), Cu^+^ has been shown to accelerate Aβ_1__–__42_ aggregation and Cu^2+^ either enhances or inhibits Aβ species aggregation in a ratio- and pH-dependent manner.^19,21,22^ Furthermore, both ROS formation and oxidative bond formation are linked processes, as copper in conjunction with H_2_O_2_ has been shown to enhance toxic Aβ dimer formation via dityrosine bridges between Aβ monomers in an oxidative process.^23^ Similarly to copper, zinc is structurally significant with the ability to bind both histidine and cysteine, e.g. in SOD1 and the large family of zinc finger domains. Zinc has distinct mechanisms for protein aggregation compared to copper but still exhibits pathological interaction with many of the same aggregation-prone peptides implicated in neurodegeneration, e.g. Aβ and tau.^11,16,20,24–26^

Interestingly, when subjected to copper toxicity, cells rapidly initiate the unfolded protein to protect against protein aggregation.^12^ This toxicity leads to cell death that is induced through the aggregation of lipoylated tricarboxylic acid cycle proteins by excess copper ions in a process denoted as *cuproptosis*.^27^ Zinc toxicity is more poorly understood than copper toxicity but is related to mismetalation of enzymes that effectively disable them as well as binding to thiol groups. Zinc toxicity also involves ROS and protein aggregation, such as for RNA-binding proteins.^28–31^ We therefore chose to use metal ion toxicity as a model in combination with direct protein aggregation assays to investigate our candidate compound, tripeptide glycyl-l-histidyl-l-lysine (GHK) for its anti-aggregative and cytoprotective abilities.

We selected the endogenous copper-binding GHK due to its ability to bind copper, zinc, and some other divalent metal ions, its presence in human blood where it may be transported around the body, and its high accumulation in the central nervous system (CNS), since it is found highest in the brain second only to the kidneys after intravenous injection in mice.^32,33^ The role of GHK is to regulate intracellular copper transport and depending on time and tissue concentration, it can either inhibit or facilitate bioactive intracellular copper levels.^34^ This modulatory role is likely a natural mechanism to fine-tune the intracellular copper levels that, among many functions, can facilitate proliferation or differentiation through loading of copper-dependent enzymes, especially COX4.^34,35^ This natural function of GHK to modulate tissue copper levels may be a way to chelate or deliver copper in a tissue/time-specific manner and thus, when trying to regulate copper levels, presents some advantages over synthetic copper chelators such as d-penacillamine that only functions as a chelator and has known toxicity issues.^36^

The scope of the study therefore focuses on GHK with its protective role in copper and, to a lesser extent, zinc-induced cell death, ROS generation, and protein aggregation, with some comparisons between other divalent metal ions. In addition to this, we investigated the protective effect of GHK *in vitro* in two more contexts that involve copper, namely the inflammatory immune response and exposure to the environmental toxin paraquat (PQ). The results obtained herein demonstrate the broad applicability of GHK to limiting the detrimental activity of these metal ions under various conditions.

## Materials and methods

### Chemicals

See Table S1.

### Cell culture

#### Primary cerebellar neurons

The protocol used was a simplified version of the following protocol, with the omission of the arabinofuranoside treatment.^37^ Male or female C57BL/6 mouse pups (Day 5–7 postnatal) were euthanized with isoflurane, and their heads removed and placed in ice-cold Hanks Balanced Salt Solution (HBSS). Cerebella were dissected and meninges carefully removed and washed twice with HBSS before dissociating with a scalpel in a Petri dish with approximately 1–2 ml of HBSS. This minced solution was then added to a Falcon tube and allowed to precipitate for 5 min, after which the remaining supernatant was removed and the remaining cerebellum fragments were incubated in 5 ml of 37°C trypsin–EDTA (0.05%) + 0.2 mg DNase for 15 min, shaking the suspension every 5 min. The supernatant was then carefully removed from the solution and 5 ml of dissociation media (L-15 media, 15% fetal calf serum (FCS), 0.2 mg/ml DNase, and 2.4 mM MgCl_2_) was added and the cells briefly spun at 200 × *g* for 5 s to gently precipitate the cells, after which the media was removed. The pellet was then resuspended in 2 ml of HBSS, 15% fetal calf serum (FCS), 0.2 mg/ml DNase, and 2.4 mM MgCl_2_ (HFD mix) and dissociated with a 100-µl pipette tip, followed by further dissociating using another 100-µl pipette tip with the mouth narrowed by pinching the plastic together using flat tweezers. The suspension was then filtered using a 100-µm filter and washed with cold HBSS. The resulting mix was then centrifuged for 10 min, 110 × *g*, 4°C, and resuspended with NB-K media [NB media, B27 supplement 1:50 dilution, KCl (22 mM), GlutaMAX 2 mM, and 1% penicillin/streptomycin] before plating in 96-well plates (coated with 0.01% poly-l-lysine for 24 h) at a density of 6 × 10^4^ cells per well.

#### Primary microglia and astrocytes

Male or female C57BL/6 mice between 3 and 5 months of age were euthanized under isoflurane and transcardially perfused with ice-cold phosphate buffered saline (PBS) until the lungs and liver were completely cleared of blood, after which the whole brains were then extracted and placed into ice-cold HBSS. HBSS was then removed and the preparation vigorously mashed with a 10-ml pipette until reduced to a pulp, after which enzymatic digestion solution (L-15 medium with papain 1:100 dilution filtered with a 0.2-μm filter) was added at a volume of 5 ml per brain and incubated for 10 min at 37°C in a water bath. The digested material was then resuspended with a 10-ml pipette until the pulp was reduced to a smooth consistency, after which 0.2 mg/ml DNase was added and the solution incubated for another 10 min at 37°C in a water bath. The digested solution was then transferred to a 50-ml Falcon tube and the enzymatic digestion quenched with 20 ml of cold HBSS and passed through a 40-µm filter and topped up with an additional 10 ml of HBSS. The filtrate was then centrifuged for 5 min at 400 × *g*, 4°C, after which the supernatant was carefully removed with a 10-ml pipette and resuspended in 37% Percoll solution (HBSS 62%, HBSS 10 × 1%, and Percoll 37%) and centrifuged for 10 min, with acceleration set at speed 4 with no brake at 800 × *g*, 4°C. The supernatant including the top myelin layer was carefully removed, and the remaining mixed glial pellet resuspended in cold HBSS and centrifuged for 5 min at 400 × *g*. The resultant pellet was then resuspended in complete DMEM/F-12 (DMEM/F-12 supplemented with 1% penicillin/streptomycin, 5 mM sodium pyruvate, 5 mM l-glutamine, and 0.2 mM 2-mercaptoethanol), 20 ng/ml M-CSF, and 10% FCS and added to T-75 flasks at 20 ml per flask, with ∼1.5 brains per flask. Culture media was refreshed every 3 days until Days 14–16 when the culture had reached ∼90% cell confluence. Cells were then detached with trypsin–EDTA (0.05%) for 10 min at 37°C in an incubator and then quenched with 10 ml of cold HBSS and centrifuged for 5 min at 400 × *g*, 4°C. To separate the microglia from the astrocytes, the pellet was resuspended in 1 ml of MACS^®^ Separation Buffer per 1-ml Falcon tube (one 15-ml Falcon tube per flask) and incubated with 40 µl of CD11b (Microglia) magnetic MicroBeads at 4°C in the dark for 30 min, after which 3 ml of MACS^®^ Separation Buffer was added. LS magnetic separation columns were pre-run with 3 ml of MACS^®^ Separation Buffer, after which the incubated cells were added under the magnetic separator. Cells collected via negative selection in the flow-through were considered primarily astrocytes. The positive selected microglia were flushed and collected in a separate tube away from the magnetic separator with 5 ml of MACS^®^ Separation Buffer. Cells were then centrifuged for 5 min at 400 × *g*, 4°C, and the microglia were resuspended in complete DMEM/F-12 with FCS and M-CSF as described for the initial mixed glial cell culture and the astrocytes cultured in complete DMEM/F-12 with FCS without MCS-F. Cells were allowed to rest in new T-75 flasks for 24 h before subsequent plating and experimentation. All astrocytes were plated at a density of 1.5 × 10^4^ cells and microglia at 2 × 10^4^ per well in 96-well plates.

#### Primary macrophages

Male or female C57BL/6 mice between 3 and 5 months of age were euthanized under CO_2_ and had their femurs extracted and cleaned before placing in cold PBS. Before bone marrow extraction, femurs were washed in 70% ethanol briefly for 30 s and again in PBS. The femurs were cut at the ends and flushed with 10 ml of PBS using a 10-ml syringe and 27 G needle to collect the bone marrow in a 15-ml Falcon tube. The bone marrow was then homogenized by trituration through the syringe until no more fragments remained in solution, which was then topped up with PBS and centrifuged for 5 min at 350 × *g*. The remaining pellet was then resuspended in 5 ml of ACK buffer for 10 min, after which PBS was added to quench the reaction, before centrifugation for 5 min at 350 × *g*. The remaining pellet was resuspended in DMEM high glucose supplemented with 1% penicillin/streptomycin, 5 mM sodium pyruvate, 5 mM l-glutamine, 0.2 mM 2-mercaptoethanol, 10% FCS, and 10 ng/ml M-CSF in a T-175 flask with four femurs per flask for a total volume of 25 ml per flask. This culture refreshed with media as stated before at Day 3 and cells harvested between Days 7 and 9 at an ∼90% confluence. All macrophages were plated at a density of 2 × 10^4^ per well in 96-well plates.

#### BV2 microglia

BV2 microglia were cultured in 20 ml of DMEM/F-12 supplemented with 1% penicillin/streptomycin, 5 mM sodium pyruvate, 5 mM l-glutamine, 0.2 mM 2-mercaptoethanol, and 10% FCS in T-75 flasks and used from Passages 1 to 4 before restarting the culture. All BV2 microglia were plated at a density of 2 × 10^4^ cells per well in 96-well plates.

### Cell counting

Cell counting was performed using the Bio-Rad TC20™ Cell counter using the 6–20 µm size inclusion range without trypan blue staining for all cell types.

### Metal ion toxicity and GHK test

Each metal ion investigated was dissolved in Milli-Q^®^ water to a concentration of 500 mM before dilution to the desired concentration between 0 and 500 µM. GHK was dissolved in PBS to a concentration of 50 mM, aliquoted, and frozen until use. All cells cultured were plated into 96-well plates at a density of 2 × 10^4^ cells per well, except for primary cerebellar neurons plated at a density of 6 × 10^4^. All cells were given at least 24 h to rest after plating to adhere before treatment with differing concentrations of metal ions in their respective growth media (DMEM/F-12 or DMEM high glucose) without FCS to limit cell growth for the duration of assay, with the exception of cerebellar neurons for which complete NB-K media was used. Each condition was prepared separately in an empty 96-well plate and then added rapidly to the cells in order to reduce pretreatment bias, to keep incubation times as close as possible, and to reduce time cells spent outside the incubator. Each assay was conducted with 200 µl of total volume per well, after which the supernatant was divided into 100 µl for the *N,N*-dimethyl-*p*-phenylenediamine dihydrochloride (DMPD) ROS assay and 100 µl for the nitric oxide (NO) inflammation assay. The remaining cells were subject to the neutral red cell viability assay, thereby generating three readouts per well. Amino acid–free media (AAFM) used for incubation was based around a recipe that consisted of the following: NaCl 145 mM, KCl 5.4 mM, CaCl_2_ 1.8 mM, MgCl_2_ 1 mM, Na_2_HPO_4_ 0.8 mM, d-(+)-glucose 5 mM, and HEPES (4-(2-hydroxyethyl)-1-piperazineethanesulfonic acid) 20 mM, pH 7.4,^38^ with an additional 0.2 mM 2-mercaptoethanol, 1% penicillin/streptomycin, and 5 mM sodium pyruvate (to better imitate cell culture media while omitting amino acids) all dissolved in Milli-Q^®^ water and then filter sterilized through a 0.2-µm filter; each assay was conducted in 100 µl of incubation media.

### ROS determination using DMPD

ROS generation was assessed using a modified DMPD method.^39,40^ In brief, a stock solution of 100 mM DMPD was prepared in Milli-Q^®^ water and 1 ml of this was added to 100 ml of 0.1 m acetic acid, pH 5.25, to generate a working solution of 1 mM. A 100 µl of this solution was added to each supernatant and incubated for 1 h at 37°C in an incubator in complete darkness. The absorbance was then read using the SpectraMax^®^ 384 Plus plate reader using SoftMax Pro 7 software with the 5-s pre-shake function and absorbance set at 505 nm.

To test the capacity of each metal ion to generate ROS in the absence of cells, the experiment was prepared exactly as described previously using DMEM/F-12 as the solvent to better replicate the conditions of cell culture in a 96-well plate. A 100 µl 1 mM DMPD solution was added to each supernatant and incubated for 1 h at 37°C in an incubator in complete darkness. The absorbance was then read using the SpectraMax^®^ 384 Plus plate reader using SoftMax Pro 7 software with the 5-s pre-shake function and absorbance set at 505 nm. Blank and control wells were performed with four technical replicates minimum and each experimental well with three.

### NO determination using the Greiss reagent

Supernatants were subject to the Greiss test to measure nitrite levels following the protocol suggested by ThermoFisher Scientific^®^ (G-7921). In brief, the crystals of *N*-(1-naphthyl)ethylenediamine dihydrochloride, sulfanilic acid, and phosphoric acid were dissolved at a ratio of 1 g to 25 ml of Milli-Q^®^ water to provide the 1× working solution. Equal volumes of this 1× solution were added to the supernatants from the previous assays and the plates kept in the dark for 15 min, after which the absorbance was read using a SpectraMax^®^ 384 Plus plate reader using SoftMax^®^ Pro 7 software with the 5-s pre-shake function and absorbance set at 540 nm. Blank and control wells were performed with four technical replicates minimum and each experimental well with three. Results were tabulated using GraphPad Prism 8 software.

### Cell viability test using neutral red

**Fig. 1:**
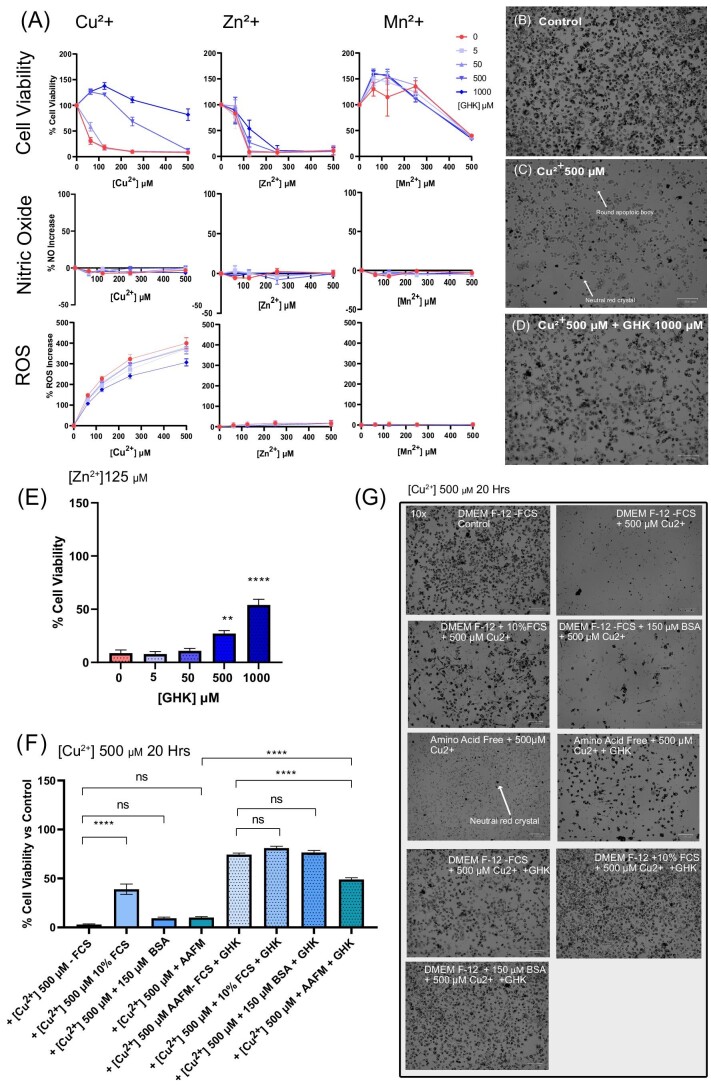
Effects of metal ions on BV2 microglia cell viability, reactive oxygen species (ROS) and nitric oxide (NO) generation, and mediation by glycyl-l-histidyl-l-lysine (GHK). (A) Cell viability of BV2 cells after incubation for 24 h with metal ions at concentrations ranging from 0 to 500 µM: each metal ion (Cu^2+^: *n* = 3, Zn^2+^: *n* = 3, and Mn^2+^: *n* = 2) is listed by column and each assay [cell viability, NO, and ROS] is listed by row. (B–D) Representative phase-contrast image of BV2 cells incubated for 24 h with (B) DMEM/F-12 without fetal calf serum (FCS), (C) 500 µM Cu^2+^, and (D) 500 µM Cu^2+^ + GHK 1000 µM. (E) *n* = 3, cell viability of BV2 cells incubated with 125 µM Zn^2+^ and treated with GHK tabulation from (A). (F) *n* = 2, cell viability of BV2 cells after incubation for 20 h with 500 µM Cu^2+^ in the presence or absence of 10% FCS, 1% (150 µM) bovine serum albumin (BSA), or in amino acid–free media (AAFM), each condition with or without 1000 µM GHK. (G) Representative phase-contrast images of (F). Each well image was stained with neutral red. All error bars are represented as SEM, with three technical replicates for (A) and four technical replicates for (F). The number of repeats pooled denoted by *n* = *x*. Not significant (ns) *P* > 0.05, *P < 0.05, **P < 0.01, ***P < 0.001, ****P < 0.0001 denoted where applicable.

The neutral red cell viability protocol served as the basis.^41^ Briefly, supernatants were removed from wells and respective cells were incubated in 100 μl of 40μg/ml neutral red containing media corresponding to the cell type being examined for 3 h at 37°C in 5% CO_2_ and 95% humidity, after which the supernatant was aspirated and plates washed twice with 100 µl PBS 1× for 5 min. A 100 µl of neutral red destain solution containing 50% of 95% ethanol, 1% glacial acetic acid, and 49% Milli-Q^®^ water of was added to the empty wells. The plates were tapped gently by hand and then placed on a flat shaker at 200 rpm (2.2g) for at least 10 minutes to reach full dye extraction from each well. Plates were then read using a SpectraMax^®^ 384 Plus plate reader using SoftMax^®^ Pro 7 software with the 5-s pre-shake function and absorbance set at 540 nm. Experiments in Figs. 1E and S1E and F were read using exactly the same settings with a SpectraMax^®^ iD3 plate reader using SoftMax^®^ Pro 7.0.3 software. Results were calculated by first subtracting each well from the average of all blank wells and then the results from each experimental condition were normalized to the average of the live control wells that themselves served as the 100% cell viability baseline. Representative images of live cells that had stained with neutral red were taken at ×10 magnification using a Bio-Rad^®^ ZOE fluorescent cell imager after 3 h of incubation with neutral red and one wash with PBS using the phase-contrast setting. Blank and control wells were performed with four technical replicates minimum and each experimental well with three.

### Direct antioxidant activity assessed using DMPD^•+^

A working solution of DMPD was obtained as previously described in the ROS determination using DMPD section. The radical cation DMPD^•+^ was generated by adding ferric chloride solution to generate a 0.1 mM final concentration in 1 mM of DMPD and left for 10 min. Stock solutions of GHK, 50 mM l-histidine (129 mM) or l-ascorbate (100 mM), were directly added to each well in 96-well plates to reach the final concentrations, and this was incubated in darkness at 37°C for 1 h, after which the absorbance was then read using the SpectraMax^®^ 384 Plus plate reader using SoftMax^®^ Pro 7 software with the 5-s pre-shake function and absorbance set at 505 nm.

### Bovine serum albumin metal ion aggregation assay

Bovine serum albumin (BSA) was dissolved to a concentration of 15 mg/ml in Tris–HCl pH 7.4 and plated in 96-well plates in 100 μl volumes. Since the molecular weight of the 583-amino-acid-long BSA is 66 430 g/mol, a concentration of 15 mg/ml is equivalent to 225 μM.^42^ The addition of 5 mM CuCl_2_ to induce aggregation represents a 22:1 ratio of Cu^2+^ to BSA and likewise a 44:1 ratio at 10 mM CuCl_2_. Next, 500 mM stock solutions of each metal ion (Cu^2+^, Zn^2+^, Mn^2+^, and Cd^2+^) were directly added to the solutions to arrive at the experimental concentrations. Each well was then allowed to incubate for the described times. GHK (1000 mM) stock in Tris–HCl pH 7.4 buffer was then also added directly to the wells for the appropriate experiments to the correct concentrations. Turbidity was assessed using the SpectraMax^®^ 384 Plus plate reader using SoftMax Pro 7 software with the 5-s pre-shake function and absorbance set at 405 nm. Supplementary experiments in Figs. S3 and 6D and F were read using exactly the same settings with a SpectraMax^®^ iD3 plate reader using SoftMax^®^ Pro 7.0.3 software. Heat-induced aggregation of BSA was carried out using an Applied Biosystems^®^ 2720 thermocycler using 96-well polymerase chain reaction (PCR) tubes with a cycle of 75°C for 20 min and then rapidly cooling to 4°C to ensure controlled heating before cooling and final transfer to a flat-bottomed clear 96-well plate for reading. Representative phase-contrast images were taken at ×10 magnification using a Bio-Rad^®^ ZOE fluorescent cell imager with the phase-contrast setting. Representative 96-well plate images were taken using a Samsung Galaxy S5 smartphone camera.

### Fluorescent copper staining via copper sensor 3

Copper levels in cells were determined using the fluorescent copper sensor 3 (CS3). Cells were plated in 96-well plates and after experimentation were first fixed in paraformaldehyde (PFA) 2% in PBS for 10 min before staining with 5 µM CS3 in 100 µl PBS for 5 min and then washing with PBS. Whole-well fluorescent intensity was then assessed using a SpectraMax^®^ iD3 microplate reader using the fluorometry function set at 530-nm excitation and 647-nm emission, as this setting gave the highest fluorescence intensity using SoftMax^®^ Pro 7 software. Representative images were taken at ×10 magnification using a Bio-Rad ZOE fluorescent cell imager prior, using the red channel with an excitation of 556/20 nm and emission of 615/61 nm.

### Immunofluorescent staining—dihydrolipoamide acetyltransferase

Cells were cultured in 96-well flat-bottom plates and after treatment were first washed with PBS before fixing with 2% PFA for 10 min at room temperature, followed by blocking with 3% BSA 0.3% Triton X-100 for 2 h. This was then followed by incubation with anti-dihydrolipoamide acetyltransferase (DLAT) antibodies diluted at a ratio of 1:200 in 100 µl of blocking buffer and incubation overnight at 4°C in darkness. The next day, cells were washed with PBS briefly and incubated with a 2° antibody conjugated with an Alexa 647 secondary antibody diluted to 1:400 in blocking buffer and incubated for 2 h at room temperature. Nuclei were stained using Hoechst 33342 after this second staining step. Cells were imaged using a Zeiss LSM 880 confocal laser scanning microscope. Each well was imaged from the center at ×10 magnification in a 2 × 2 tile and excited with a 633-nm laser and read at 638–755 nm for DLAT and excited with a 405-nm laser for Hoechst 33342 read at 410–507 nm. Images were then exported and analyzed using Fiji (ImageJ) software. For counting cells, the Hoechst channel images were thresholded to indicate the nuclei and automatically counted using the analyze particle function. For the DLAT image channel, individual thresholding was performed in each experiment, after which the remaining high-intensity particles above a threshold were considered DLAT-positive aggregates, counted using the analyze particle function. The ratio between the DLAT-positive aggregates/cell was then calculated.

### Inflammation assays

Cells were incubated in 96-well plates in complete DMEM/F-12 without FCS, and lipopolysaccharide (LPS) (100 ng/ml) and interferon gamma (IFN-γ) (20 µg/ml) were added for 24 h. Cells were then incubated with neutral red in complete DMEM/F-12 without FCS for 3 h, after which cell viability was determined as mentioned earlier. Representative images were taken at ×10 magnification using a Bio-Rad ZOE fluorescent cell imager using the phase-contrast setting.

### Copper-enhanced PQ toxicity

BV2 cells were cultured in 96-well plates at a density of 2 × 10^4^ cells per well. PQ diluted to 100 µM in complete DMEM/F-12 media without FCS was then added to the cells and left to incubate for 24 h either with or without Cu^2+^ between 0 and 500 µM or with or without GHK. Cells were then subject to neutral red testing as described earlier, and representative images were taken at ×10 magnification using a Bio-Rad ZOE fluorescent cell imager with the phase-contrast setting. Neutral red intensity was analyzed with Fiji (ImageJ) software taking the integrated intensity of each image.

### Statistical analysis

All graphs were tabulated and statistically analyzed using GraphPad Prism 8.0 software. Data are presented as mean ± SEM; all experiments have been conducted in duplicate or triplicate unless otherwise stated and where possible are pooled. For each figure, the type of statistical analysis, *P* value ranges, sample sizes, and number of pooled experiments are given. For single variable experiments, Student's *t*-tests were performed and for experiments with multiple variables, one-way analysis of variance (ANOVA) tests were used. The threshold for statistical significance was considered if *P* < 0.05. Significance levels (*) were indicated as *P < 0.05, **P < 0.01, ***P < 0.001, and ****P < 0.0001.

### Graphics

Graphical abstract was created using BioRender software.

### Ethical statement

All experiments involving the use of mice or cells/tissue derived from mice were conducted in accordance with the ethical permit Dnr: 9328-2019 approved by the Stockholm Animal Research Ethics Board, Sweden. No other ethical permission was required for the other experiments described.

## Results and discussion

### Cell viability, NO, and ROS levels after divalent metal ion exposure and modulation by GHK

In order to first establish the ability of GHK to modulate metal ion toxicity, three metal ion species were chosen, namely Cu^2+^, Zn^2+^, owing to their aforementioned participation in neurodegenerative diseases and the ability of GHK to form complexes with both, and also Mn^2+^ due to its involvement in α-synuclein aggregation related to PD, for which the interaction with GHK has not been well documented.^43,44^ BV2 microglia were chosen as the preliminary cell type for these investigations as they are easily cultured and functionally recapitulate most aspects of primary microglia.^45^ Furthermore, aside from astrocytes, microglia play a vital role in the brain homeostasis of metal ions in the CNS and also in control of the inflammatory environment, which in turn greatly affects the homeostasis of metal ions.^46^

In order to first establish the relative toxicities of each metal ion, BV2 microglia were exposed to titrated concentrations of Cu^2+^, Zn^2+^, and Mn^2+^ in the presence/absence of GHK. Determination of the protective effect of GHK on cytotoxicity was the primary aim, followed by the effect of GHK on ROS level changes induced by these metal ions (as ROS generation is a major factor in cell death) and also the inflammatory response as measured by NO production due to the major role that inflammation plays in metal ion homeostasis.^47–49^ As depicted in Fig. 1A, different metal ions exhibited different inherent toxicity levels, with the most toxic being both Cu^2+^ and Zn^2+^, followed by Mn^2+^, which displayed detectable cytotoxicity up to 500 µM. The observed toxicities could be effectively attenuated by GHK in a concentration-dependent manner for Cu^2+^ at each concentration and at 125 μM for Zn^2+^ (Fig. 1 A and E), with no apparent effect on Mn^2+^. Visual effects of 500 µM copper toxicity on BV2 cells were evident as these dead cells were present with roughly spherical bodies without uptake of neutral red, indicating nonviable cells (Fig. 1C). This death was prevented by GHK, with living cells being able to retain neutral red similar to controls (Fig. 1B vs Fig. 1D). As a control experiment, GHK alone was tested for general effects on cell viability in the absence of metal ion toxicity as this has been previously reported for hepatocytes and could possibly be a factor contributing to improved cell viability.^50^ In this manner, GHK seems to increase BV2 cell viability in a dose-dependent manner without metal ion toxicity (Fig. S1A) and may therefore contribute in part to the cytoprotective effects seen in Fig. 1. Bar chart representations outlining statistical tests of key aspects of Fig. 1A are presented in Fig. S1B–D.

With regard to ROS, Cu^2+^ induced the greatest increase, which could be attenuated by GHK (Fig. 1A). However, neither Zn^2+^ nor Mn^2+^ had any detectable effect on ROS production. This was expected as neither Zn^2+^ nor Mn^2+^ is a redox active metal, but Cu^2+^ readily participates in redox reactions to produce ROS.^51^ GHK was able to attenuate this ROS increase in a dose-dependent manner. It was unable to fully prevent ROS increase, even at a concentration of up to 1000 µM [GHK] vs 500 µM [Cu^2+^], a 2:1 ratio. Although GHK has been reported to render Cu^2+^ redox inactive upon complexation, one study has contested this finding, showing that the GHK–Cu complex does still form ROS in solution, albeit significantly lower than Cu^2+^ alone, which corroborates our findings.^52,53^

For the inflammatory response, none of the tested metals induced an increase in NO production. There are contradictory reports in the literature for Cu^2+^, as one study using BV2 cells indicated an increase in NO production following Cu^2+^ incubation^54^ while another study reported that Cu^+^ could inhibit LPS-induced NO production *in vitro*, suggesting an anti-inflammatory role.^55^ Our results here show no evidence of NO increase with BV2 microglia by Cu^2+^ stimulation alone.

One important factor that must be controlled for is the amino acid composition of the cell culture media in understanding the effect of GHK on Cu^2+^ and Zn^2+^ toxicity since GHK is able to form ternary complexes with other amino acids and proteins such as serum albumins.^56,57^ This confounding factor was partially controlled for by the omission of FCS that contains a variable amount of growth factors and other proteins and minerals that could interact with both metal ions or GHK.^58^ Furthermore, using DMEM/F-12 as the basis for the incubation media posed an issue as it contains around 20 different amino acids often in the range of 2.5–0.05 mM, some of which GHK could form ternary complexes with, especially histidine and cysteine.^57,59,60^ We therefore investigated both Cu^2+^ and Zn^2+^ toxicity and the protective effect of GHK in different media compositions, which included DMEM/F-12 with or without the addition of FCS (10%), BSA (1%, equivalent to 10 mg/ml or 150 µm), or with amino acid free media (AAFM).

AAFM with 500 µM Cu^2+^ resulted in almost total loss of cell viability after 20 h, but the addition of 1000 µM GHK to the media could partially rescue this, although it was still lower than with GHK in the presence of DMEM/F-12 without FCS (Fig. 1E and F). Incubation with 10% FCS alone for 20 h also gave a protective effect against Cu^2+^ toxicity that was not evident after 24 h of Cu^2+^ incubation (Fig. 1E vs Fig. S1D). For BSA, however, at 20 h of 500 µM Cu^2+^ incubation and despite no significant protective effect evident in the quantitative neutral red assay, live cells could still be observed by phase contrast, suggesting a slight protective effect of BSA for Cu^2+^ (Fig. 1F and G). This protective effect of GHK in AAFM was also the same for zinc toxicity, as following 125 µM Zn^2+^ incubation for 24 h, the protective effects of FCS, BSA, and GHK both alone and in synergy were recorded (Fig. S1E and F). This confirms the protective effect of GHK, even in the absence of amino acids in the media, and that GHK works in synergy with other amino acids to achieve the effects seen in Fig. 1A–D.

One limitation we noted was with the neutral red spectrophotometric method of cell viability, namely that AAFM + 500 µM Cu^2+^ in Figs. 1F, G, and S1D, all show some low level of cell viability, despite no evidence of live cells accumulating neutral red by phase-contrast images. We believe this is due to the rapid toxicity of Cu^2+^ in the absence of amino acids and the preservation of the cells that have died this way *in situ*, since even 1 h of +500 µM Cu^2+^ in AAFM led to total cell death, but with the cell bodies and morphology clearly preserved (Fig. S2A–D). The remnant cell bodies would provide nonspecific retention of neutral red dye or crystal fragments that would not be present in apoptotic cells as they detach and are washed away (Fig. S2D), therefore providing an artificially high neutral red viability signal for AAFM + 500 µM Cu^2+^.

Overall, these initial findings using BV2 microglia yielded some insights into the protective effects of GHK on metal ion–induced toxicity. However, cell lines are inherently imperfect models and BV2 microglia only represent one cell type of the CNS, and we therefore next sought to recapitulate the key findings using primary neurons, microglia, and astrocytes in order to confirm the results obtained.^61^

### Protective effect of GHK on copper and zinc toxicity in primary CNS cell cultures

**Fig. 2:**
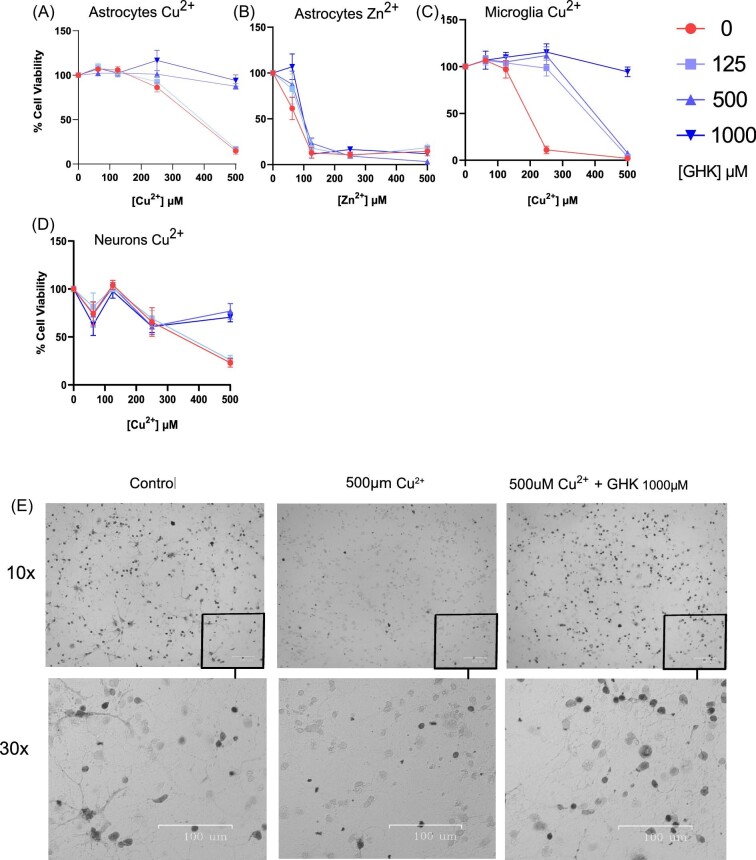
Effects of metal ions on primary central nervous system (CNS) cell viability and mediation by glycyl-l-histidyl-l-lysine (GHK). (A, B) Cell viability of primary astrocytes incubated with increasing concentrations of (A) Cu^2+^, (B) Zn^2+^ ranging from 0 to 500 μM for 24 h with GHK concentrations ranging from 0 to 1000 μM (*n* = 2). (C) Cell viability of primary microglia incubated with increasing concentrations of Cu^2+^ ranging from 0 to 500 μM for 24 h, with GHK concentrations ranging from 0 to 1000 μM (*n* = 1). (E) Cell viability of primary cerebellar neurons from C57BL/6 mice incubated with increasing concentrations of (D) Cu^2+^ ranging from 0 to 500 μM for 24 h, with GHK concentrations ranging from 0 to 1000 μM (*n* = 2). (H) Representative phase-contrast images of primary cerebellar neurons incubated in 0–500 μM Cu^2+^ and GHK, top row ×10 magnification, bottom row digital ×30. All error bars are represented as SEM, with three technical replicates. The number of experiments pooled in graph denoted by *n* = *x*.

Since Cu^2+^ and Zn^2+^ were the most cytotoxic to BV2 cells and that this outcome could be modulated by GHK, we decided to test these effects on other CNS cells from C57BL/6 mice. Aside from recapitulating the effects in primary microglia, astrocytes were also chosen due to their important role in CNS metal ion homeostasis and neurons as the key cells of interest in neurodegenerative diseases, with cerebellar neurons being chosen as a high purity neuronal model.^37,62^ Firstly, astrocytes were subject to Cu^2+^ and Zn^2+^ toxicity with/without GHK, and similar effects were observed as those for BV2 microglia (Fig. 2A vs Fig. 1A). The effect of Cu^2+^ on cell viability was recapitulated in primary microglia (Fig. 2B). Finally, Cu^2+^ toxicity was evaluated in primary cerebellar neurons, which demonstrated a significant decrease in cell viability at 500 µM that could also be rescued by GHK (Fig. 2D). Microscopic examination revealed neurons with cell bodies positively stained for neutral red in the GHK-treated group subject to Cu^2+^ toxicity, similar to the controls but with less neutral red accumulation in the axons (Fig. 2E). GHK also had no difference on neuronal viability, demonstrating its lack of cytotoxicity (Fig. S1H).

GHK offers protective effects from metal ion toxicity for Cu^2+^ and Zn^2+^ demonstrated in both cell lines and primary cultures, although we have not fully extended these findings to Zn^2+^ toxicity in primary microglia or neurons. What remains to be understood is the mechanism by which this effect is achieved. One of the mechanisms of copper toxicity is its ability to generate ROS directly via redox cycling between its 1^+^ and 2^+^ states that can generate ROS; we demonstrate GHK's ability to partially prevent this in Fig. 1A.^14^ Although a necessary part of cellular functions, uncontrolled excess ROS induces DNA damage, protein and enzyme damage, lipid peroxidation, and mitochondrial dysfunction.^63^ Another mechanism of copper toxicity is by the aforementioned participation in protein aggregation, which could work in conjunction with this redox cycling capacity of copper in the catalysis of pathological disulfide bond formation, enzyme inactivation, and protein aggregation.^64^ GHK was able to attenuate Cu^2+^-induced cytotoxicity, possibly through either of these mechanisms (Figs. 1 and 2).

Apart from Cu^2+^ toxicity, we also show that GHK prevents Zn^2+^ toxicity in astrocytes. The mechanism of action of Zn^2+^ toxicity is not fully understood but involves entry into the cells through channels shared by iron and calcium, and this is not suppressed *in vitro* by free radical scavengers.^65^ This indicates a lack of direct ROS formation as a mechanism of toxicity, which is supported by our observation that Zn^2+^-induced cytotoxicity does not increase ROS generation and that Zn^2+^ is known to be catalytically inert.^66,67^ However, Zn^2+^ has been demonstrated to induce ROS indirectly *in vivo*, via the interaction between Zn^2+^ and NADPH oxidase in ischemic brain injury in rats, whereby excess zinc promotes a positive feedback loop that causes excessive ROS production via NADPH oxidase activation, further Zn^2+^ accumulation and neuronal cell death that can be attenuated with use of a Zn^2+^ chelator.^68^ The DMPD assay is unlikely to be sensitive enough to detect these intracellular changes that happen indirectly and thus we did not observe any free radical generation in Fig. 1A.

Zn^2+^ toxicity is also implicated in other neurological diseases aside from ischemic stroke such as traumatic brain injury (TBI), PD, and AD. For instance, neuronal injury forces release of Zn^2+^ from presynaptic terminals that rapidly accumulates in postsynaptic neurons, leading to rapid death, and the chelation of zinc has been demonstrated to reduce neuronal death during ischemia, TBI, and seizures by up to 85%.^2,69,70^ One study reported that GHK was effective in preventing astrocytic and neuronal injury in a mouse model of intracerebral hemorrhage, although the effects on zinc were not studied.^71^ Considering the differences and similarities in the mechanism of action of Cu^2+^ and Zn^2+^ toxicity, GHK was nonetheless able to protect CNS cells under these circumstances.

### Antioxidant evaluation of GHK

**Fig. 3:**
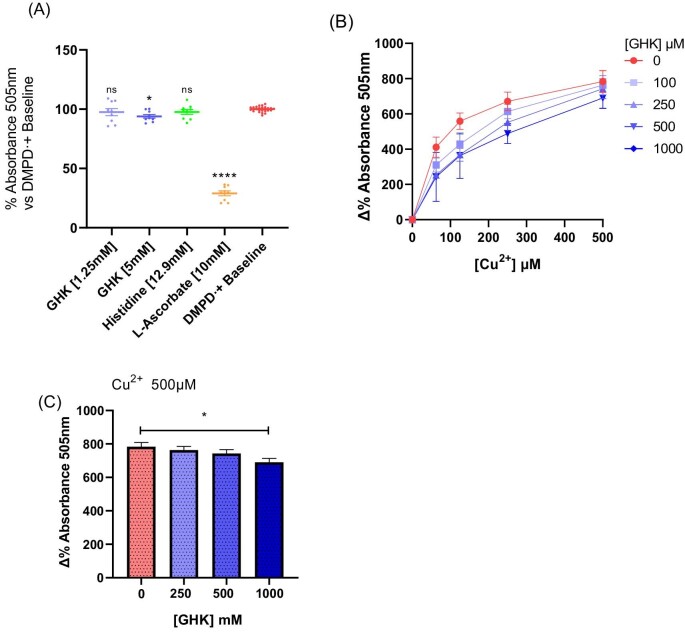
Glycyl-l-histidyl-l-lysine (GHK) prevents copper-mediated reactive oxygen species (ROS) production but with limited direct antioxidant ability. (A) Dimethyl-*p*-phenylenediamine dihydrochloride radical cation (DMPD^•+^) incubation with GHK, histidine, and l-ascorbate for 1 h (*n* = 3). (B) ROS-mediated oxidation of DMPD via Cu^2+^ in the absence of cells and its inhibition by GHK (*n* = 2). (C) Bar chart representation of data taken from (C) 500 μM Cu^2+^ + GHK 0–1000 μM (*n* = 2). All error bars are represented as SEM, with three technical replicates. Not significant (ns) *P* > 0.05, *P < 0.05, **P < 0.01, ***P < 0.001, ****P < 0.0001 denoted where applicable. The number of experiments pooled in graph denoted by *n* = *x*.

The high elevation of ROS production from Cu^2+^ incubation and the fact that GHK reduced Cu^2+^ redox inactivity (Fig. 1A) initially led us to believe that ROS was the major cause of cell death and that the protective effect we observed with GHK was its inhibition. To better understand the antioxidant ability of GHK, we used the DMPD^•+^ assay to assess if GHK displayed any direct antioxidant abilities. In this regard, l-ascorbate was used as a positive control to demonstrate complete reduction of the DMPD^•+^ radical and histidine to investigate antioxidant ability of the central residue of GHK. Histidine has been reported as a free radical scavenger that would aid in the interpretation of which amino acid moieties if any conferred antioxidant ability.^39,72^ Although GHK has been previously described to act as a direct antioxidant for hydroxyl radicals, it did not exhibit any ability to reduce DMPD^•+^ to DMPD^+^ at 1.25 mM, a concentration close to that used in our cell culture conditions (1 mM) (Fig. 3A).^73^ Furthermore, histidine at 12.9 mM (over 2.5× molar excess compared to GHK) also had no effect. This result demonstrates that neither GHK nor histidine can act as an antioxidant H^+^ donor to any significant degree. However, at 5 mM, GHK did have a very minor yet statistically significant antioxidant effect and is thus unlikely to be responsible for the observed cytoprotective effect. The minor effect may possibly be from the antioxidant effects of both histidine and lysine in combination.^74^

Next, to evaluate whether the generated ROS were due to the metal ions themselves or were produced through cell stress, we repeated the incubation with Cu^2+^ in the complete absence of cells. ROS were still generated (Fig. 3B and C), demonstrating the oxidative potential of the metal ion itself as the primary generator of ROS, which was attenuated by GHK. This demonstrates the mechanism of how GHK prevents ROS by acting directly to prevent metal ion species participation in ROS generation. Interestingly, GHK cannot completely eliminate the radical generation despite being in molar excess. However, since Zn^2+^ caused an even higher degree of cell death, but had no ability to catalyze ROS formation by conversion of DMPD to the DMPD^•+^ free radical (Fig. 1A), we believe the effect may also be due to an additional mechanism of the toxicity of these metals through which GHK worked in a protective capacity, namely protein aggregation.

### GHK prevents copper-induced DLAT aggregation

**Fig. 4:**
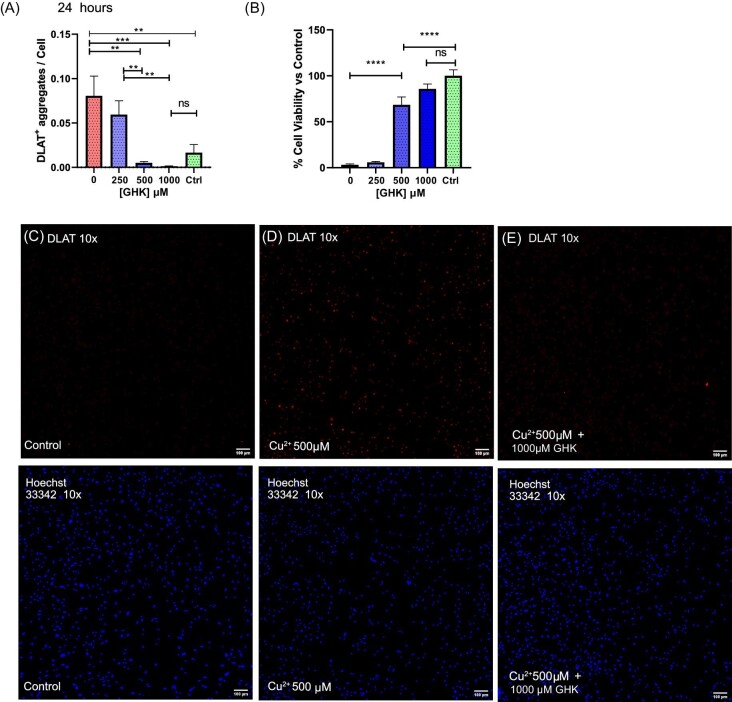
Glycyl-l-histidyl-l-lysine (GHK) prevents copper toxicity–induced dihydrolipoamide *S*-acetyltransferase (DLAT) aggregation in macrophages. (A, B) *n* = 2. Twenty-four hours of incubation with 500 μM Cu^2+^ and GHK 0–1000 μM: (A) DLAT aggregates per cell; (B) cell viability. (C–E) Representative images of DLAT^+^ aggregates per cell after 24 h of copper exposure: (C) control, (D) 500 μM Cu^2+^, (E) 500 μM Cu^2+^ + GHK 0–1000 μM. All error bars are represented as SEM, with four technical replicates. Not significant (ns) *P* > 0.05, *P < 0.05, **P < 0.01, ***P < 0.001, ****P < 0.0001 denoted where applicable. The number of experiments pooled in graph denoted by *n* = *x*.

To investigate whether GHK functioned to prevent protein aggregation *in vitro*, we cultured primary bone marrow macrophages, due to their functional similarity to primary microglia, in order to confirm the cause of cell death, namely cuproptosis. We thus evaluated the copper-induced aggregation of lipoylated DLAT, a hallmark of cuproptosis, and observed that 500 µM Cu^2+^ induced an increase in DLAT^+^ aggregate-containing cells after 24 h and that this was almost completely reduced in a concentration-dependent manner by GHK (Fig. 4A and E).^75^ This was inversely related to the cell viability, with higher cell viability correlating with decreased DLAT^+^ aggregates (Fig. 4A vs B).

### GHK prevents intracellular accumulation of Cu^+^

**Fig. 5:**
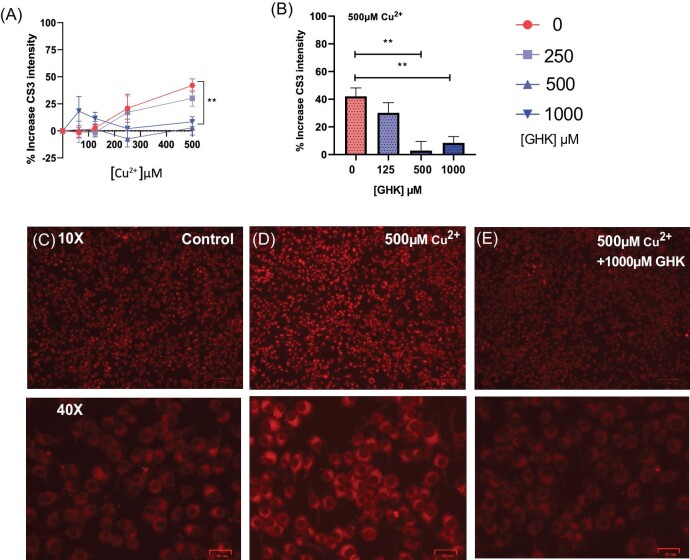
Glycyl-l-histidyl-l-lysine (GHK) prevents Cu overloading in BV2 cells under conditions of toxic Cu excess. (A) Changes in BV2 cell intracellular copper levels after 1 h of incubation in Cu^2+^ concentrations ranging from 0 to 500 μM, with GHK concentrations ranging from 0 to 1000 μM (*n* = 2). (B) Bar chart representation of data taken from (A) *n* = 2, 500 μM Cu^2+^ with varying concentrations of GHK. (C–E) Representative images of BV2 cells after 1 h of incubation, with varying concentrations of Cu^2+^ and stained with copper sensor 3 (CS3) at ×10 (top) and ×40 (bottom) magnifications: (C) control; (D) 500 µM Cu^2+^; (E) 500 µM Cu^2+^ + GHK 1000 µM. All error bars are represented as SEM, with three technical replicates. Not significant (ns) *P* > 0.05, *P < 0.05, **P < 0.01, ***P < 0.001, ****P < 0.0001 denoted where applicable. The number of experiments pooled in graph denoted by *n* = x.

The mechanism by which GHK prevents Cu^2+^ toxicity has not yet been established, but we believe it may be due to its putative alternative action to inhibit copper entry into the cell.^34^ To investigate this, we incubated BV2 microglia with Cu^2+^ for 1 h to monitor the early stages of Cu^2+^ toxicity using the fluorescent copper stain, CS3, a BODIPY-based exogenous labile Cu^+^ probe that is highly selective for Cu^+^ over Cu^2+^ and other metal ions.^76^ A 500 μM Cu^2+^ incubation led to a marked increase in CS3 fluorescence intensity, indicating increased intracellular levels of Cu^+^, and this could be prevented by GHK in a dose-dependent manner (Fig. 5A, B, and D). It is possible that GHK is acting by sequestering the copper in its Cu^2+^ form extracellularly and thus preventing its conversion into Cu^+^, import via copper transporter 1 (CTR1) and thus detection intracellularly by CS3.^77^ Alternatively, it is also possible that GHK accumulates intracellularly as it is hypothesized to enter in complex with Cu^2+^ but would therefore still be unable to be detected by CS3.^33^ However, since GHK forms ternary complexes with GSH extracellularly to facilitate the reduction of Cu^2+^ to Cu^+^ for import via CTR1, the buffering of GHK in the extracellular compartment is probable, given this existing function.^60^ We have not managed to elucidate this aspect of GHK's copper overload prevention, but it possibly works via both mechanisms as they are not mutually exclusive.

Although GHK is best known for its ability to promote copper transport intracellularly, we have demonstrated its ability to prevent Cu^+^ accumulation in Fig. 5.^34^ The reasons for this ability of GHK have been suggested before, as high levels of GHK alone without Cu greatly stimulate stem cell proliferation, whereas GHK-Cu increases cell differentiation.^78^ This is likely related to low Cu levels being known to promote cell proliferation via metabolic reprogramming toward a more glycolytic metabolism, whereas adequate copper loading promotes mitochondrial complex 4 assembly and promotes oxidative metabolism and cell differentiation.^35^

The question of which compartment GHK exerts its Cu sequestering properties in remains unclear. Clues may lie in the histidine-copper metabolism, since high levels of histidine can similarly induce functional copper deficiency but high total intracellular copper levels through direct intracellular buffering.^79^ Furthermore, experiments with rat hypothalamic slices indicate that although excess histidine (1000× over copper) could prevent copper uptake, copper complexation to histidine and other amino acids was necessary for brain copper uptake in a process that suggests copper and histidine do not enter as a conjugate but are in fact both taken up separately after dissociation.^80^ This is relevant to GHK, as the histidine residue is the primary copper-binding peptide in GHK, and it is possible that GHK may behave in a similar manner to promote the effect we have seen here. However, the additional amino acids surrounding histidine may confer it additional receptor affinities and effector functions as it putatively binds to the transforming growth factor beta (TGFβ) family of receptors.^33^ Furthermore, other potential transporters with which GHK and Cu may form complexes include l-type amino acid transporter 1 (LAT1/SLC7A5), which has been recently demonstrated to shuttle the Cu(His)_2_ intracellularly, and the peptide-histidine transporter 1/2 (PHT1/2), which also transports small peptides across the blood–brain barrier.^81,82^ Future studies could be undertaken to investigate such receptors that would enhance our understanding of the mechanisms by which GHK transports or blocks transport of Cu.

### GHK prevents and reverse copper- and zinc-induced BSA aggregation

Copper and zinc are known to both directly participate in protein aggregation and induce Aβ formation *in vitro*, as well as being highly concentrated in AD senile plaques.^83,84^ This effect on enhancing protein aggregation also includes tau, α-synuclein, islet amyloid polypeptide in Type 2 diabetes and AD, and also β-crystallins in cataract formation.^11,85–87^ We therefore examined the aggregation potential of different divalent metal ions Cu^2+^, Zn^2+^, Mn^2+^, and Cd^2+^ to recapitulate previous findings regarding the unique abilities of Cu^2+^ and Zn^2+^ in protein aggregation and the effect of GHK as a modulator. We chose BSA as a model protein to investigate the anti-aggregative effects of GHK. Despite BSA being highly ordered, lacking self-aggregation properties, and being without a protease-resistant core, it does possess the ability to form beta sheets with many reported mechanistic similarities to Aβ1–40 fibrillation, and is thus a suitable protein for investigations of protein aggregation.^88–90^ When Cu^2+^ reacts with BSA at 1:4 and 1:16 ratios, aggregates form immediately and increase in a time-dependent manner that corresponds to an increasingly negative charge.^91^ This is also the case in our experiments, as immediate addition of the respective metal ions at the experimental concentrations resulted in BSA solution turbidity (data not shown).

**Fig. 6:**
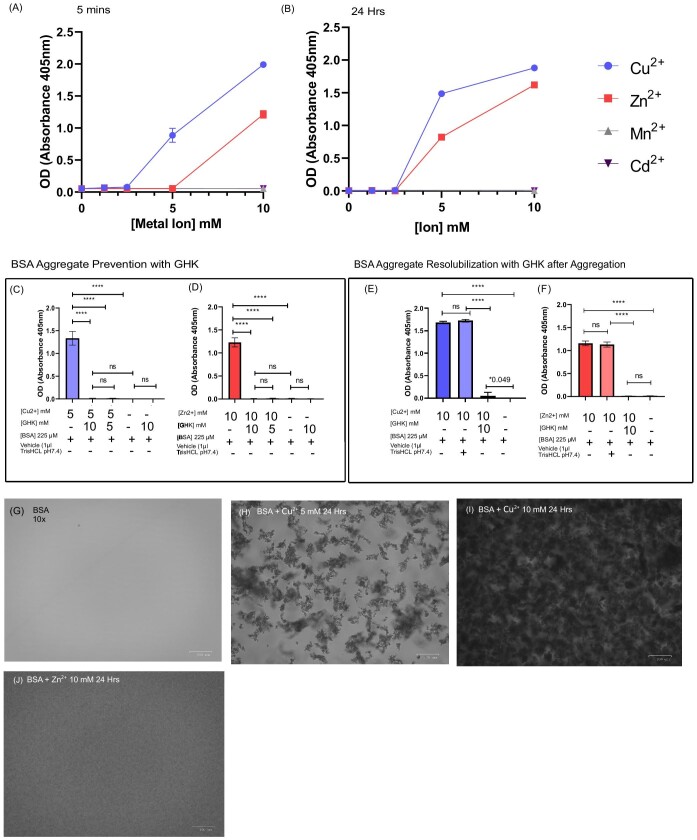
Metal ion–mediated albumin aggregation and its inhibition and reversal by glycyl-l-histidyl-l-lysine (GHK). (A, B) *n* = 2; 100 µl of 15 mg/ml (225 μM) bovine serum albumin (BSA) was exposed to 5 mM of Cu^2+^, Zn^2+^, Cd^2+^, and Mn^2+^ and examined for aggregation at different time points: (A) 5 min, (B) 24 h, (C) *n* = 2, BSA pretreated with 5 or 10 mM GHK and incubated with 5 mM Cu^2+^ for 5 min. (D) *n* = 3, BSA pretreated with 5 or 10 mM GHK and then incubated with 5 mM Zn^2+^ for 5 min and examined for aggregation. (E) *n* = 2, BSA incubated in 10 mM Cu^2+^ for 5 min to allow aggregate formation, followed by the addition of GHK at 10 mM and a further 5 min of incubation before examining for aggregation. (F) *n* = 3, BSA incubated in 10 mM Zn^2+^ for 5 min to allow aggregate formation, followed by the addition of GHK at 10 mM and a further 5 min of incubation before examining for aggregation. (G–J) Representative phase-contrast images (×10) of Cu^2+^- and Zn^2+^-induced BSA aggregation. Vehicle corresponds to 1 µl of Tris–HCl pH 7.4 buffer. All error bars are represented as SEM. Technical replicates per experiment *n* = 4 except (A) and (B), where *n* = 3. All error bars are represented as SEM. Not significant (ns) *P* > 0.05, *P < 0.05, **P < 0.01, ***P < 0.001, ****P < 0.0001 denoted where applicable. The number of experiments pooled in graph denoted by *n* = *x*.

Only Cu^2+^ and Zn^2+^ induced a time-dependent BSA aggregation that is consistent with previous findings.^12^ Both Mn^2+^ and Cd^2+^ are known agents that induce protein aggregation *in vitro*, but in the case of BSA, we observed no direct aggregation ability (Fig. 6A and B), which is likely due to their influence on the stages of protein folding rather than direct aggregation of mature proteins.^43,92^ This ability to affect mature proteins makes both copper and zinc species particularly relevant in the aggregation of CNS proteins such as Aβ. Cu^2+^ was a stronger inducer of protein aggregation than Zn^2+^, both in terms of aggregation speed and final optical density (Fig. 6A and B). BSA aggregation induced by Cu^2+^ and Zn^2+^ was inhibited by the addition of excess GHK (Fig. 6C and D). Phase-contrast images revealed that dense aggregates were formed after 24 h of BSA exposure to 5 and 10 mM Cu^2+^ (Fig. 6H and I). In the case of exposure to 10 mM Zn^2+^, much finer structured aggregates formed compared to those of Cu^2+^ (Fig. 6K). GHK addition after Cu^2+^ and Zn^2^^+^ BSA aggregate formation could also resolubilize the BSA, leading to a reduced optical density that was almost complete at equimolar levels of GHK and Cu^2+^ and complete for Zn^2+^ (Fig. 6E and F). We have so far demonstrated that both Zn^2+^ and Cu^2+^ facilitate BSA aggregation and that Cu^2+^ is a stronger inducer of aggregation than Zn^2+^ (Fig. 6A, B, E, and F) that supports existing literature.^12,90,93^ In both cases, we observed that GHK could prevent and reverse aggregation of metal ion–induced BSA aggregation. Since aggregation can be rapidly reversed upon the addition of a chelator, the Zn^2+^ and Cu^2+^ ions do not seem to induce any permanent aggregation.

To further test this theory, we then decided to dilute preformed BSA aggregates in excess 100 μl Tris–HCl pH 7.4 buffer to halve the concentration and to examine the effect on aggregate dissolution. Dilution would serve to lower the concentration of both BSA and the metal ion that itself could resolubilize the BSA, provided it is not permanently aggregated.^94^ Our results in Fig. S3A and B show that for both Cu^2+^ and Zn^2+^, dilution by 100 μl of buffer led to a complete dissolution of the BSA aggregates for both metal ion species. Furthermore, GHK was again tested and was found to able to resolubilize the BSA, whereas the vehicle alone (1 μl buffer) showed no reduction in turbidity or aggregate dissolution (Fig. S3C and D). Further qualitative images of Cu^2+^- and Zn^2+^-induced BSA aggregation are included in Fig. S3A, B, and E. Furthermore, to determine if GHK's resolubilization of BSA is specific to this metal ion–induced aggregation, heat-induced aggregation of BSA was employed, as this type of aggregation is generally permanent.^95^ In brief, BSA was heated to 75°C for 20 min, after which GHK was tested for its resolubilization potential. In this case, the addition of GHK after the formation of these aggregates had no effect on heat-induced BSA aggregate resolubilization in contrast with Zn^2+^- or Cu^2+^-induced BSA aggregates (Fig. S3F). However, the addition of GHK to the BSA before heat treatment had a significant effect in preventing the heat-induced aggregation of BSA. This may be related to the presence of amino acids lysine and histidine, as lysine has been shown to inhibit heat-induced lysozyme aggregation and both lysine and histidine in improved protein solubility.^96–98^ Furthermore, the addition of 100 µl of buffer to BSA did not resolubilize the aggregates; however, surprisingly, dilution seemed to increase the aggregation directly after addition. This counter-intuitive result could possibly be explained by a combination of dilution increasing the propensity of BSA to aggregate by increasing the relative proportion of aggregate-prone BSA in combination with heat-induced changes to BSA speciation.^89,99^ GHK is therefore able to prevent and reverse Zn^2+^- and Cu^2+^-induced BSA aggregation, as well as to inhibit heat-induced aggregation of BSA, but is unable to reverse it.

The formation of these Cu^2+^ BSA aggregates is probably caused firstly by the saturation of Cu-binding sites on the BSA before binding nonspecifically to other exposed amino acids.^91^ GHK possibly acts by forming a chelate with the Cu^2+^ ions interacting with BSA and thereby removing them from participation in aggregation by providing an increase in available high-affinity ligands. Since BSA only consists of four metal ion–binding sites, the total canonical copper-binding maximum for the solution would be 900 μM of binding sites (BSA 225 μM × 4) that is insufficient to fully bind 5 or 10 mM of Cu^2+^ or Zn^2+^ at the high 22:1 and 44:1 Cu^2+^ to BSA ratios we have used herein.^100^ GHK used with BSA (at 5  or 10 mM) therefore offers ample binding sites for Cu^2+^ or Zn^2+^. Furthermore, GHK's ability to form ternary complexes with imidazole-containing peptides [including serum albumin's N-terminal site Asp-Ala-His (NTS)] and with GHK itself means that such ternary complexes are likely being formed under these experimental conditions that also may affect aggregation kinetics.^101,102^ One study has demonstrated that a 5 mM concentration of GHK in conjunction with HSA and Cu^2+^ leads to an increase in the prevalence of both binary Cu-(GHK) and ternary Cu-(GHK)N_IM_^GHK^ complexes that together form more than 40% of the detected species, the rest being predominantly the Cu-(GHK)N_IM_^HSA^ ternary complex, with a fraction being solely bound to HSA.^56^ It would be possible that even a greater ratio of GHK-Cu binary and ternary complexes would be evident in this experiment as the ratio of GHK to BSA is even higher. Seeing as this is the same concentration of GHK used in this study, these data would support the idea of GHK-Cu complexes directly sequestering Cu^2+^ and preventing the BSA aggregation. In the case of Zn^2+^, GHK binding is distinct from that of Cu^2+^ but also likely acts by sequestering Zn^2+^ from its interaction with BSA.^44^ The difference in observed structural composition of the aggregates formed by Cu^2+^ or Zn^2+^could also be related to the different binding sites on the protein.^103^ Although a highly artificial model, our results show that GHK could function to help maintain the solubility of proteins under conditions of metal ion–induced aggregation.

### Inflammation increases cell toxicity to copper that can be partially rescued by GHK

**Fig. 7:**
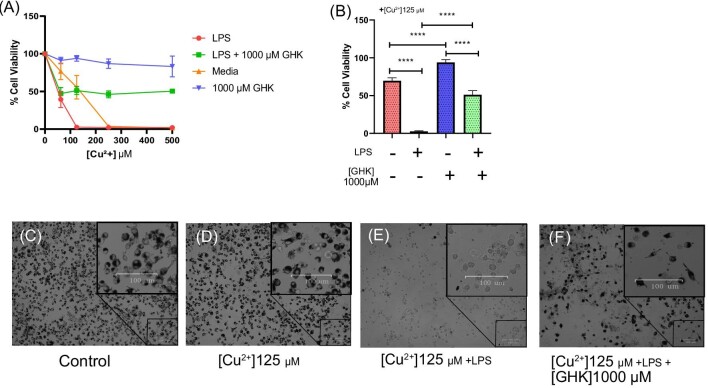
Lipopolysaccharide (LPS)–mediated sensitivity to Cu^2+^ cytotoxicity and attenuation by glycyl-l-histidyl-l-lysine (GHK). (A) Cell viability of BV2 cells incubated with increasing concentrations of Cu^2+^ ranging from 0 to 500 µM with or without the presence of 1000 µM GHK or LPS for 24 h (*n* = 2). (B) Effect of 125 µM Cu^2+^ with or without the presence of 1000 µM GHK or LPS for 24 h, taken as an excerpt from (A) (*n* = 2). (C, D) Representative phase-contrast image (×10) of BV2 cells incubated for 24 h with 125 µM Cu^2+^ with or without the presence of 1000 µM GHK or LPS, including a ×20 digital magnification on cell clusters per condition: (C) control, (D) 125 µM Cu^2+^, (E) 125 µM Cu^2+^ + LPS, (F) 125 µM Cu^2+^ + LPS + 1000 µM GHK (*n* = 2). All error bars are represented as SEM, with three technical replicates. Not significant (ns) *P* > 0.05, *P < 0.05, **P < 0.01, ***P < 0.001, ****P < 0.0001 denoted where applicable. The number of experiments pooled in the graph is denoted by *n* = *x*.

Understanding this anti-aggregative ability of GHK in relation to copper, we next sought to investigate its potential in conditions known to enhance intracellular copper influx. Our interest is the inflammatory process, as this is an important aspect of neurodegenerative diseases. As immune responses are strongly dependent on copper signaling, we expected inflammation to affect copper toxicity. We therefore treated BV2 microglia with LPS and IFN-γ, which together are a strong pro-inflammatory stimulus for myeloid cells.^104^ IFN-γ stimulation is known to increase surface CD44 expression that promotes endocytic iron and copper uptake and so we expected an increase in copper uptake.^105^ Cells pretreated for 24 h with LPS/IFN-γ and then exposed to Cu^2+^ had a significantly increased sensitivity to Cu^2+^ toxicity, and this could also be ameliorated by GHK (Fig. 7A and B). The LPS pretreatment increased the toxicity of Cu^2+^, with around 99% cell death at 125 µM Cu^2+^ compared to about 25% cell death with just Cu^2+^ alone, which could be partially ameliorated by GHK (Fig. 7B–F).

It is important to note the differences between the Cu^2+^-only group in these experiments compared to the first experimental design (Fig. 7A vs Fig. 1A), as here they were exposed to an additional 24 h without FCS. Interestingly, viability only reached a maximum of about 50% with LPS treatment regardless of Cu^2+^ or GHK concentration, suggesting a mechanism of Cu^2+^ toxicity that cannot be attenuated by GHK (Fig. 7A). Overall, GHK demonstrated cytoprotective effects on LPS-enhanced Cu^2+^ toxicity.

In agreement with our hypothesis, we report the novel finding that inflammation induces increased susceptibility to Cu^2+^-induced cell death in BV2 microglia that could again be partly ameliorated by GHK (Fig. 7). This finding may be relevant to diseases that involve both elevated copper concentrations and inflammation, a characteristic of neurodegenerative diseases such as AD and PD, but also for autoimmune diseases such as multiple sclerosis (MS) and rheumatoid arthritis.^106,107^ In the case of MS, one study reported an increase in astrocytic CTR1 expression in both patient tissues and the experimental mouse model, facilitating copper uptake and distribution in the CNS while increasing demyelination.^108^ The remaining question to answer is whether inflammatory microglia or macrophages affect other cells in the local environment such as neurons or astrocytes, thereby developing a more Cu^2+^ vulnerable state. This increased vulnerability would be due to inflammation promoting excessive cellular uptake of copper, leading to downstream protein aggregation and ultimately to cell death. This is particularly troublesome in the CNS, as the turnover of copper in the CNS is exceedingly slow (at 20–2000× less than other organs), thus presenting unique vulnerabilities to metal ion disturbances.^109^ The concentration at which Cu^2+^ induces cell death in LPS/IFN-γ–treated myeloid cells (125 µM) is close to estimated levels in the synaptic cleft (100 µM) and paired with dysfunction of the synapses in neurodegenerative diseases presents a case for inflammation-enhanced CNS copper sensitivity.^110,111^ While the effects of inflammation on zinc cytotoxicity have not yet been studied, they are also directly relevant to neuroinflammatory diseases.

### Copper exposure enhances PQ-induced toxicity

**Fig. 8:**
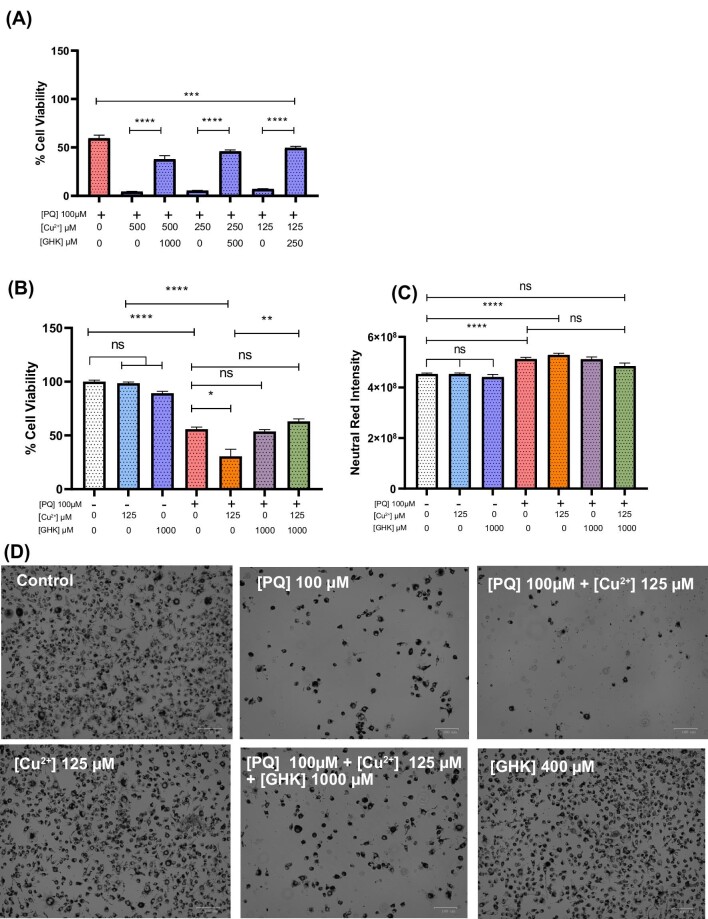
PQ-mediated sensitivity to Cu^2+^ cytotoxicity and attenuation by glycyl-l-histidyl-l-lysine (GHK). (A) Cell viability of paraquat (PQ)–treated BV2 cells with 0–500 μM Cu^2+^ and a 2:1 GHK ratio (*n* = 1). (B) Cell viability of PQ-treated BV2 cells with or without 125 µM Cu^2+^ and/or + 1000 µM GHK (*n* = 2). (C) Neutral red intensity of BV2 cells taken from experiment (B) (*n* = 2). (D) Representative phase-contrast images of BV2 cells from experiments (B, C). All error bars are represented as SEM, with four technical replicates minimum. Not significant (ns) *P* > 0.05, *P < 0.05, **P < 0.01, ***P < 0.001, ****P < 0.0001. The number of experiments pooled in graph is denoted by *n* = *x*.

Next, we sought to examine the effect of GHK in another model system of ROS generation using PQ, a known environmental toxin that has been strongly implicated in neurodegenerative diseases. In PD and ALS, exposure to PQ is strongly correlated to the incidence of these diseases in epidemiological studies and in mouse models of PD that employ PQ as a primary toxin.^112–114^ Furthermore, PQ is a known protein aggregate inducer and ALS risk factor.^115–117^ We used BV2 microglia and first titrated PQ in order to establish a toxic dose (data not included). We determined 100 µM PQ to be sufficient to induce 50% cell death and used this as a working concentration. Next, we treated BV2 cells with PQ while maintaining a 1:2 Cu to GHK ratio in order to maintain a molar excess of GHK with declining copper levels. We observed that PQ toxicity was greatly enhanced in the presence of copper and that this could be partially restored by GHK (Fig. 8A). However, GHK alone could not restore the viability of PQ-treated cells to the level of the control group, reaching a maximum of 50% (Fig. 8B). Furthermore, the surviving cells treated with PQ had an increased density of neutral red staining that is indicative of lysosomal swelling, which may be related to an autophagic process that is evident in the early stages of PQ toxicity (Fig. 8C and D).^118^

Although the mechanism of action of PQ is not fully understood, ROS generation and the reduction of antioxidant potential of the cells were described first.^115^ However, there are two previously described reactions of PQ that are either aerobic or anaerobic. The former involves the biological reduction of PQ into PQ^•+^. PQ^•+^ then reacts with O_2_ to form PQ^2+^ and O_2_^•^, the highly damaging superoxide anion, which is relevant to our experiments as the conditions are aerobic. The latter is strongly suggested to be due to PQ^2+^ reduction of Cu^2+^ to Cu^+^ under hypoxia as the main cause of toxicity. Hypoxia is known to sensitize both mammalian and bacterial cells to copper toxicity and to enhance the conversion of Cu^2+^ to Cu^+^. It is specifically the Cu^+^ oxidation state that strongly induces nonspecific aggregation of cysteine- and histidine-containing proteins and toxicity.^18,119,120^ Since separate experiments were not undertaken ensuring hypoxia, we have not been able to address the effect of GHK on this aspect of PQ toxicity. Overall, we demonstrate the increased cytotoxicity of PQ with Cu^2+^ under oxygenated conditions and extend these findings to show that Cu^2+^-enhanced toxicity can be attenuated with the addition of GHK.

Despite our results indicating enhancement of PQ toxicity with Cu^2+^ in BV2 microglia (consistent with previous findings from bacteria and primary rat cerebellar neurons, other studies conducted in yeast and SHSY-5Y neuroblastoma cells suggest that copper is protective against PQ toxicity.^119,121–123^ Our findings with BV2 microglia are more comparable with the former study involving neurons as we noted PQ toxicity derived for neuronal toxicity to be in a similar range (100 µM). It is thus evident that considering the discrepancies in reported results, copper modulation is an important factor in the toxicity of PQ. More investigation into chelators/ionophores such as GHK under different oxidative conditions to gain a better understanding of these discrepancies is warranted.

## Conclusion

Copper homeostasis is a key aspect of cell survival and a key basic biological function, acting as a redox catalyst to facilitate these important biological processes. However, its strong catalytic ability makes it extremely cytotoxic, such that one atom is estimated to be unbound in a cell.^124^ The reasons behind this are 2-fold: firstly, the redox activity promotes production of ROS that are intrinsically cytotoxic, leading to the formation of lipid peroxides and can also promote formation of oxidized thiol bonds contributing to protein aggregation.^125,126^ Secondly, direct aggregation due to the ability of copper to bind to amino acids cysteine and histidine promotes the aggregation of proteins together at these sites.^20,127^ In the case of copper toxicity, it has been established that protein aggregation is a central driver of cuproptosis as opposed to oxidative stress, a feature that is common across phyla.^12,18^ Likewise, zinc is highly toxic in its free form and drives protein aggregation through similar mechanisms by cysteine and histidine bond formation and aggregation.^86,128^

In this study, we demonstrate that GHK can prevent both Cu^2+^- and Zn^2+^-induced cell death in several major cell types relevant to the CNS. However, the mechanisms underlying this protective effect were not entirely clear. Initially, we hypothesized the ability of GHK to bind divalent metal ions and to reduce their redox activity, proving this protective effect by inhibiting ROS production. However, the slight decrease in ROS generation in comparison with the greatly enhanced cell survival of Cu^2+^ challenged cells with GHK agrees with previous findings that ROS are not the primary cause of cell death during cuproptosis, but instead it is protein aggregation. Our study highlights the utility of GHK in this inhibitory function of Cu^2+^ overload, preventing aggregation of key proteins such as DLAT (Fig. 4). GHK may therefore be useful a secondary sequestration method for Cu^2+^, in support of GSH and also metallothioneins that bind and store Cu^+^.^12,129^ Furthermore, we demonstrate the effect of LPS and IFN-γ in enhancing Cu^2+^ toxicity and the ability of GHK to prevent this, which highlights the increased vulnerability to copper that a neuroinflammation presents, and the protective role of GHK in this regard. In addition to this, we demonstrate that Cu^2+^ enhances the toxicity of PQ that could also be partially inhibited by GHK, together demonstrating the broad utility of this tripeptide in the prevention of Zn^2+^ and Cu^2+^ toxicities under different conditions.

Overall, we speculate that the relation between metal ions and neurodegeneration represents a gradual failure of amino acid–based metal ion buffering systems in the CNS as we age. For instance, GHK levels in a 20 years old is 200 ng/ml, which declines to 80 ng/ml by the time the individual becomes 60 years old; likewise, histidine and albumin see similar declines that are somewhat sex-dependent.^130–132^ This leaves a system such as the CNS with already extremely low metal ion turnover more vulnerable to fluctuations of especially zinc and copper that result from aging, inflammation, environmental toxins, and trauma that lead to protein aggregation and neurodegeneration. Since the transport of copper into the brain is strongly regulated by amino acids and small peptides, it is logical to employ some of the brain's natural mechanisms to balance this. In this way, GHK could be used additionally to or buffer copper levels in the CNS in a concentration-dependent manner.^76,133,134^ Herein, we have provided evidence that intervention with the endogenous copper-binding tripeptide GHK may be beneficial in regulating metal ions in the CNS related to protein aggregation, ROS, and cell death *in vitro* that is broadly relevant to neurodegenerative disease therapy.

## Supplementary Material

mfae019_Supplemental_Files

## Data Availability

Upon request.
