# Supplementary material for: Glycyl-l-histidyl-l-lysine prevents copper- and zinc-induced protein aggregation and central nervous system cell death in vitro
Source: Metallomics. 2024 Apr 10;16(5):mfae019. doi: 10.1093/mtomcs/mfae019 (PMC11135135; doi:10.1093/mtomcs/mfae019)
Supplement: mfae019_Supplemental_Files [file mfae019_supplemental_files.zip › All_Figure_legends_24314.docx]

**Figure Legends:**

**Main Manuscript:**

***Figure 1. Effects of metal ions on BV2 microglia cell viability, ROS and NOS generation and mediation by GHK.*** *(A) Cell viability of BV2 cells after incubation for 24 hours with metal ions at concentrations ranging from 0-500 µM: Each metal ion (Cu^2+^ n=3, Zn^2+^ n=3 and Mn^2+^ n=2) is listed by column and each assay (Cell viability, NO and ROS) is listed by row, (B-D) Representative phase contrast image of BV2 cells incubated for 24 hours with: (B) DMEM F-12 without fetal calf serum, (C) 500 µM Cu^2+^ and (D) 500 µM Cu^2+^ + GHK 1000 µM, (E) n=3 Cell viability of BV2 cells incubated with 125 µM Zn^2+^ and treated with GHK tabulation from figure 1A, (F) n=2 Cell viability of BV2 cells after incubation for 20 hours with 500 µM Cu^2+^ in the presence or absence of: 10% FCS, 1% (150 µM BSA), or in amino acid free media (AAFM), each condition with or without 1000 µM GHK. (G) Representative phase contrast images of (F). Each well imaged was stained with neutral red. All error bars are represented in SEM with 3 technical replicates for (A) and 4 technical replicates for (F). Number of repeats pooled denoted by n=x. All statistical significance was determined by one-way ANOVA, Tukey’s multiple comparison (P < 0.05*, P < 0.05*, P < 0.01**, P < 0.001***, P < 0.0001****).*

***Figure 2. Effects of metal ions on primary CNS cell viability and mediation by GHK.*** *(A,B) Cell viability of primary astrocytes incubated with increasing concentrations of (A) Cu^2+^,(B) Zn^2+^ from 0-500 μM for 24 hours with GHK concentrations ranging from 0-1000 μM n=2. (C) Cell viability of primary microglia incubated with increasing concentrations of Cu^2+^ from 0-500 μM for 24 hours with GHK concentrations ranging from 0-1000 μM n=1. (D-G) Cell viability of primary cerebellar neurons from C57/Bl6 mice incubated with increasing concentrations of (D) Cu^2+^ from 0-500 μM for 24 hours with GHK concentrations ranging from 0-1000 μM n=2. (H) Representative phase contrast images of primary cerebellar neurons incubated in 0-500 μM Cu^2+^ and GHK, top row 10x magnification, bottom row digital 30x. All error bars are represented in SEM with 3 technical replicates statistical significance determined by one-way ANOVA, Tukey’s multiple comparison (P < 0.05*, P < 0.05*, P < 0.01**, P < 0.001***, P < 0.0001****), number of experiments pooled in graph denoted by n=x*

***Figure 3. GHK prevents copper-mediated ROS production but with limited direct antioxidant ability.*** *(A) DMPD^•+^ radical cation incubation with GHK, histidine and L-Ascorbate for 1 hour. n=3 (B) ROS-mediated oxidation of DMPD via Cu^2+^ in the absence of cells and its inhibition by GHK. n=2 (C) Bar chart representation of data taken from (C) 500 μM Cu^2+^ + GHK 0-1000 μM n=2. All error bars are represented in SEM with 3 technical replicates, statistical significance determined by one-way ANOVA, Tukey’s multiple comparison (P < 0.05*, P < 0.05*, P < 0.01**, P < 0.001***, P < 0.0001****), number of experiments pooled in graph denoted by n=x.*

***Figure 4. GHK prevents copper toxicity-induced DLAT aggregation in macrophages.*** *(A,B) n=2. 24 hours incubation with 500 μM Cu^2+^ and GHK 0-1000 μM, (A) DLAT aggregates per cell. (B) Cell viability. (C-E) Representative images of DLAT^+^ aggregates per cell after 24 hours of copper exposure, (C) Control, (D) 500 μM Cu^2+^, (E) 500 μM Cu^2+^ + GHK 0-1000 μM. All error bars are represented in SEM with 4 technical replicates, statistical significance determined by one-way ANOVA, Tukey’s multiple comparison (P < 0.05*, P < 0.05*, P < 0.01**, P < 0.001***, P < 0.0001****), number of experiments pooled in graph denoted by n=x.*

***Figure 5. GHK prevents Cu overloading in BV2 cells under conditions of toxic Cu excess.*** *(A) Changes in BV2 cell intracellular copper levels after 1 hour of incubation in Cu^2+^ ranging from 0 to 500 μM with GHK concentrations ranging from 0-1000 μM n=2. (B) Bar chart representation of data taken from (A) n=2, 500 μM Cu^2+^ with varying concentrations of GHK. (C-E) Representative images of BV2 cells after 1 hour of incubation with varying concentrations of Cu^2+^ and stained with CS3 at 10x (top) and 40x (bottom) magnifications. (C) Control. (D) 500 µM Cu^2+^. (E) 500 µM Cu^2+^ + GHK 1000 µM. All error bars are represented in SEM with 3 technical replicates statistical significance determined by one-way ANOVA, Tukey’s multiple comparison (P < 0.05*, P < 0.05*, P < 0.01**, P < 0.001***, P < 0.0001****), number of experiments pooled in graph denoted by n=x.*

***Figure 6. Metal ion-mediated albumin aggregation and its inhibition and reversal by GHK.*** *(A**,B) n=2 100 µl of 15 mg/ml (225 μM) BSA was exposed to 5 mM of Cu^2+^, Zn^2+^, Cd^2+^ and Mn^2+^ and examined for aggregation at different time points: (A) 5 minutes, (B) 24hrs, (C) n=2 BSA pretreated with 5 or 10 mM GHK and incubated with 5 mM Cu^2+^ for 5 minutes. (D) n=3 BSA pretreated with 5 or 10 mM GHK then incubated with 5 mM Zn^2+^ for 5 minutes and examined for aggregation. (E) n=2 BSA incubated in 10 mM Cu^2+^ for 5 minutes to allow aggregate formation followed by addition of GHK at 10 mM and a further 5 minutes incubation before examining for aggregation. (F) n=3 BSA incubated in 10 mM Zn^2+^ for 5 minutes to allow aggregate formation followed by addition of GHK at 10 mM and a further 5 minutes incubation before examining for aggregation. (G-J) Representative phase contrast images (10X) of Cu^2+^- and Zn^2+^-induced BSA aggregation. Vehicle corresponds to 1µl of TrisHCL 7.4 buffer, All error bars are represented in SEM, technical replicates per experiment n=4 except figures A&B where n=3, number of experiments pooled in graph denoted by n=x, All error bars are represented in SEM, statistical significance determined by one-way ANOVA, Tukey’s multiple comparison (P < 0.05*, P < 0.05*, P < 0.01**, P < 0.001***, P < 0.0001****), number of experiments pooled in graph denoted by n=x.*

***Figure 7. LPS-mediated sensitivity to Cu^2+^ cytotoxicity and attenuation by GHK****. (A) Cell viability of BV2 cells incubated with increasing concentrations of Cu^2+^ from 0-500 µM with or without the presence of 1000 µM GHK or LPS for 24 hours n=2. (B) Effect of 125 µM Cu^2+^ with or without the presence of 1000 µM GHK or LPS for 24 hours, taken as an excerpt from figure A n=2. (C-D) Representative phase contrast image (10X) of BV2 cells incubated for 24 hours with 125 µM Cu^2+^ with or without the presence of 1000 µM GHK or LPS, including a 20x digital magnification on cell clusters per condition: (C) Control, (D) 125 µM Cu^2+^, (E) 125 µM Cu^2+^ + LPS, (F) 125 µM Cu^2+^ + LPS + 1000 µM GHK . (G-H) n=2. All error bars are represented in SEM, number of experiments pooled in graph denoted by n=x. All error bars are represented in SEM with 3 technical replicates statistical significance determined by one-way ANOVA, Tukey’s multiple comparison (P < 0.05*, P < 0.05*, P < 0.01**, P < 0.001***, P < 0.0001****), number of experiments pooled in graph denoted by n=x.*

***Figure 8. Paraquat-mediated sensitivity to Cu^2+^ cytotoxicity and attenuation by GHK.*** *(A) Cell viability of PQ treated BV2 cells with 0-500 μM Cu^2+^ and a 2:1 GHK ratio. n=1 (B) Cell viability of PQ treated BV2 cells with or without 125 µM Cu^2+^ and/or + 1000 µM GHK n=2. (C) Neutral red intensity of BV2 cells taken from experiment (B) n=2. (D) Representative phase contrast images of BV2 cells from experiment (B-C). All error bars are represented in SEM with 4 technical replicates minimum, statistical significance determined by one-way ANOVA, Tukey’s multiple comparison (P < 0.05*, P < 0.05*, P < 0.01**, P < 0.001***, P < 0.0001****), number of experiments pooled in graph denoted by n=x.*

***Supplementary Figure Legends:***

***Fig S1. Supplementary BV2*** ***Microglia and Neuron Data. (A)*** *n=3* *Cell viability of BV2 cells incubated for 24 hours with GHK concentrations ranging from 0-1000 µM. (B) n=3 Cell viability of BV2 cells incubated with GHK for 24 hours in DMEM F-12 media without FCS with 500 µM Cu^2+^ tabulation from figure 1A. (C) n=3 ROS generation of BV2 cells incubated with 250 µM Cu^2^+ and treated with GHK tabulation from figure 1A. (D) n=2 Cell viability of BV2 cells after incubation for 24 hours with 500 µM Cu^2+^ in the presence or absence of: 10% FCS, 1% (150 µM BSA), or in amino acid free media (AAFM), each condition with or without 1000 µM GHK. (E) 24 hours with 125 µM Zn^2+^ in the presence or absence of: 10% FCS, 1% (150 µM BSA), or in amino acid free media (AAFM), each condition with or without 1000 µM GHK. (F) Representative phase contrast images of (E), (G) n=2 Cell viability of primary C57 B/6 mouse cerebellar neurons incubated with 500 µM Cu^2+^ for 24 hours and treated with GHK tabulation from figure 2D.,(H) n=2 Cerebellar neurons incubated with 0-1000 µM GHK for 24 hours, All error bars are represented in SEM with 3 technical replicates (A,B,C,D,E,H) 4 technical replicates (E,F), Statistical significance was determined by one-way ANOVA, Tukey’s multiple comparison (P < 0.05*, P < 0.05*, P < 0.01**, P < 0.001***, P < 0.0001****). Number of experiments pooled in graph denoted by n=x.*

***Fig S2. Effects of amino acid free media (AAFM) on Cu^2+^ toxicity*** ***after 1 hours and 24 hours using BV2 microglia cells*** *(A, C) Cell viability of BV2 cells after incubation for (A) n=2 1 hour with 500 µM Cu^2+^ in AAFM (C) Representative phase contrast images of (A) stained with neutral red. (B,D) Cell viability of BV2 cells after incubation for (B) n=2 24 hours in AAFM alone (D) Representative phase contrast images comparing cells incubated for 24 hours in AAFM with 500 µM Cu^2+^(left) vs DMEM-F12 –FCS +500 µM Cu^2+^(right) stained with neutral red, all error bars are represented in SEM with 4 technical replicates minimum. Statistical significance was determined by one-way ANOVA, Tukey’s multiple comparison (P < 0.05*, P < 0.05*, P < 0.01**, P < 0.001***, P < 0.0001****). Number of experiments pooled in graph denoted by n=x.*

***Figure S3. Resolubilization of protein aggregates formed by Cu^2+^ and Zn^2+^ by buffer dilution (A-B****) n=2, 100 μl of 15mg/ml (225μM) BSA was exposed to (A) 5 mM of Cu^2 +^ or (B) 10 mM of Zn^2+^ and allowed to aggregate for 15-30 minutes before addition of 100 μl TrisHCl 7.4 buffer and then re-examined for aggregation after 10 minutes. (C,D) n=2 A repeat of (A,B) where 100 μl of 225μM BSA was exposed to (C) 5 mM of Cu^2 +^ or (D) 10 mM of Zn^2+^ and allowed to aggregate for 15-30 minutes before addition of 100 μl TrisHCl 7.4 buffer, 10 mM GHK in* *1μl of TrisHCl 7.4 buffer (vehicle) or vehicle and then re-examined for aggregation after 5 minutes, (E) representative images of wells of BSA under different conditions, (F) n=3 BSA heated at 75°C for 20 minutes under different conditions then examined for aggregation within 15 minutes. BSA+GHK+Cu^2+^ was performed by forming Cu-BSA aggregates first then adding GHK to dissagregate. All error bars are represented in SEM, technical replicate per experiment n=4, number of experiments pooled in graph denoted by n=x, All error bars are represented in SEM with 3 technical replicates statistical significance determined by one-way ANOVA (P < 0.05*, P < 0.05*, P < 0.01**, P < 0.001***, P < 0.0001****), number of experiments pooled in graph denoted by n=x.*
