# Supplementary figures and images for: Glycyl-l-histidyl-l-lysine prevents copper- and zinc-induced protein aggregation and central nervous system cell death in vitro
Source: Metallomics. 2024 Apr 10;16(5):mfae019. doi: 10.1093/mtomcs/mfae019 (PMC11135135; doi:10.1093/mtomcs/mfae019)

S1.

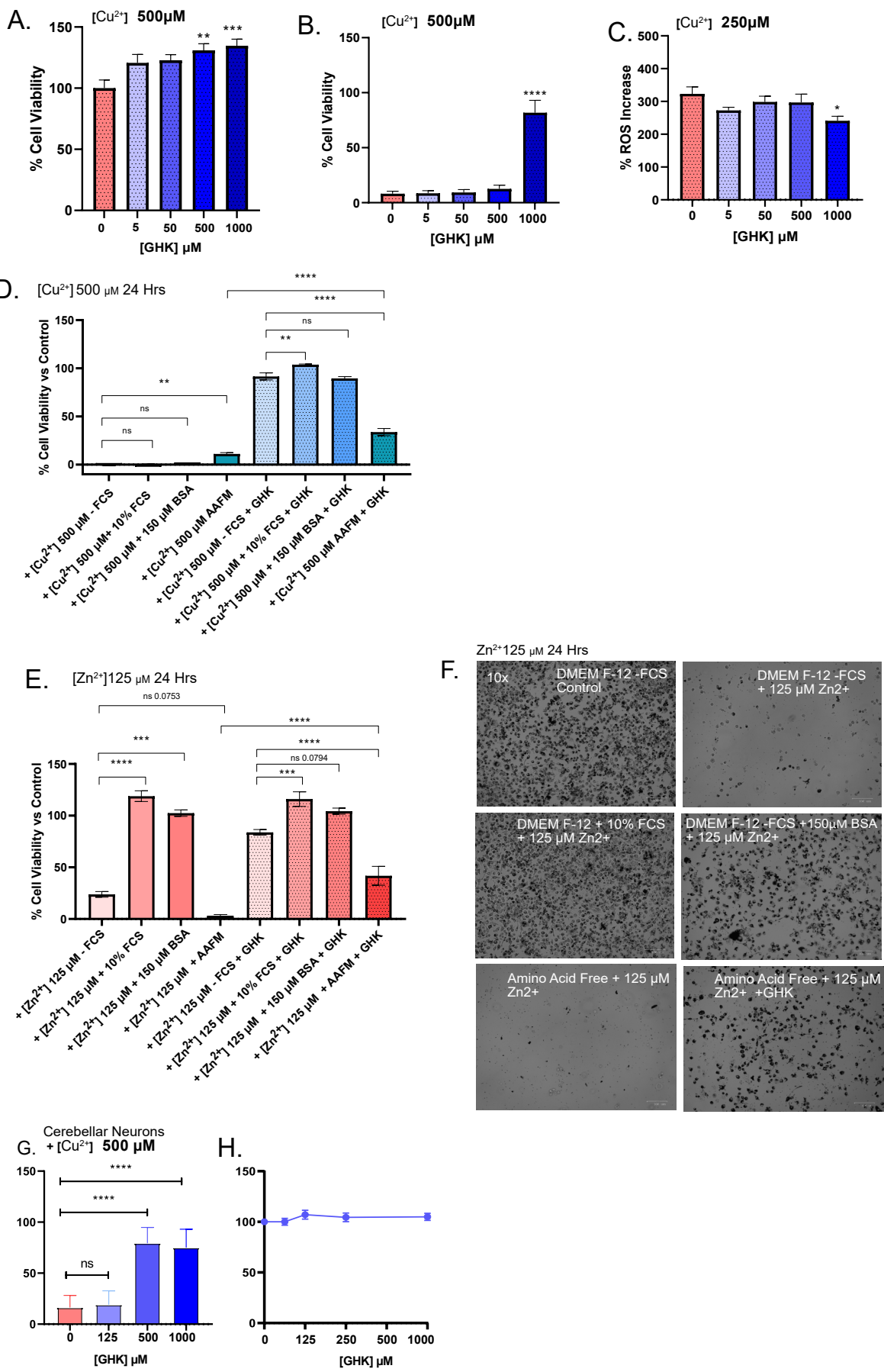

Supplement: mfae019_Supplemental_Files [file mfae019_supplemental_files.zip › Suppl_data_Figure_S1_240320.pdf]

Figure S2.

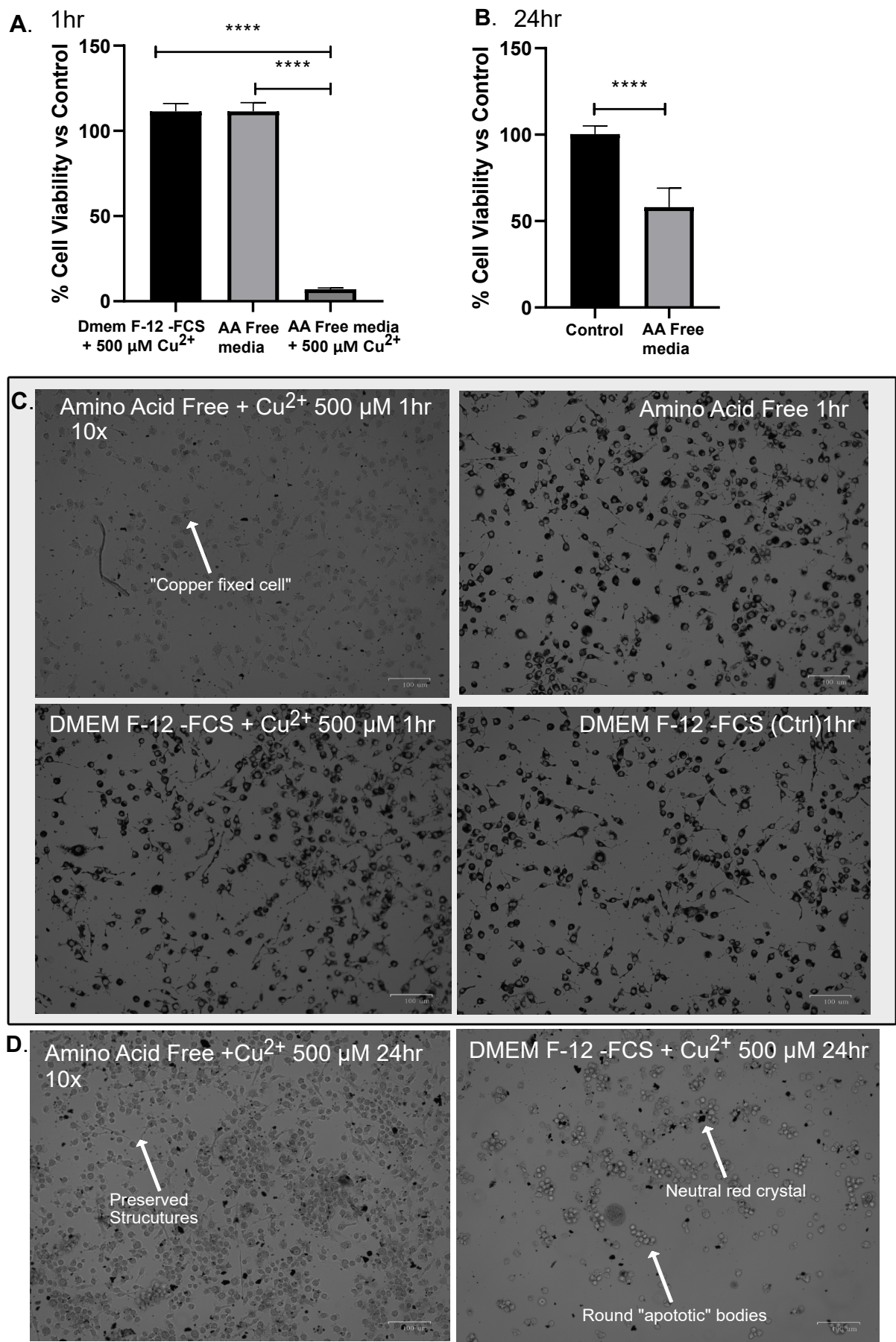

Supplement: mfae019_Supplemental_Files [file mfae019_supplemental_files.zip › Suppl_data_Figure_S2_240320.pdf]

Figure S3.

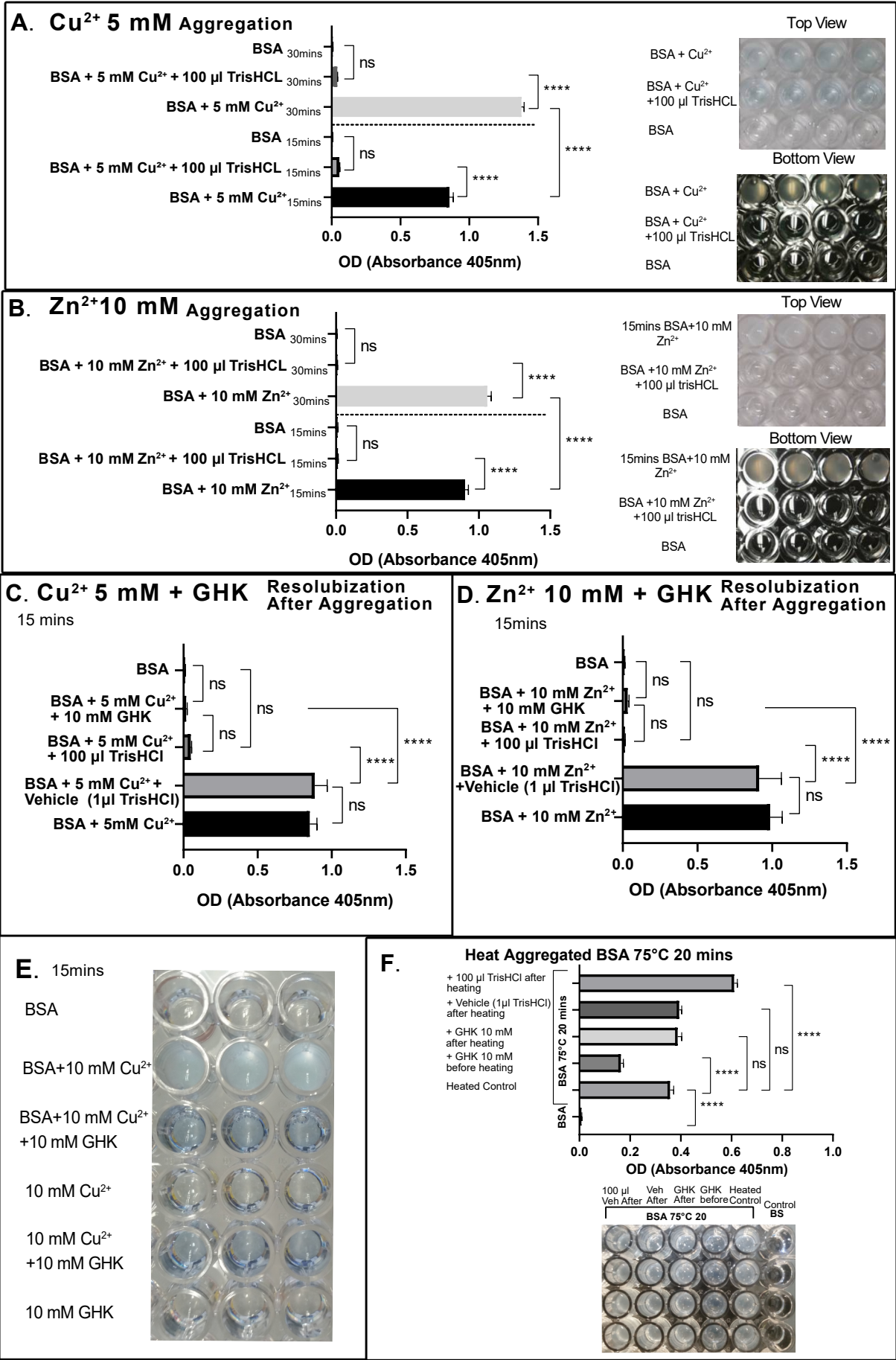

Supplement: mfae019_Supplemental_Files [file mfae019_supplemental_files.zip › Suppl_data_Figure_S3_2403220.pdf]
